# Darwin’s agential materials: evolutionary implications of multiscale competency in developmental biology

**DOI:** 10.1007/s00018-023-04790-z

**Published:** 2023-05-08

**Authors:** Michael Levin

**Affiliations:** 1 Allen Discovery Center at Tufts University, 200 Boston Ave. 334 Research East, Medford, MA 02155 USA; 2 Wyss Institute for Biologically Inspired Engineering at Harvard University, 3 Blackfan St., Boston, MA 02115 USA

**Keywords:** Embryogenesis, Regeneration, Competency, Intelligence, Problem-solving, Morphogenesis, Evolutionary

## Abstract

A critical aspect of evolution is the layer of developmental physiology that operates between the genotype and the anatomical phenotype. While much work has addressed the evolution of developmental mechanisms and the evolvability of specific genetic architectures with emergent complexity, one aspect has not been sufficiently explored: the implications of morphogenetic problem-solving competencies for the evolutionary process itself. The cells that evolution works with are not passive components: rather, they have numerous capabilities for behavior because they derive from ancestral unicellular organisms with rich repertoires. In multicellular organisms, these capabilities must be tamed, and can be exploited, by the evolutionary process. Specifically, biological structures have a multiscale competency architecture where cells, tissues, and organs exhibit regulative plasticity—the ability to adjust to perturbations such as external injury or internal modifications and still accomplish specific adaptive tasks across metabolic, transcriptional, physiological, and anatomical problem spaces. Here, I review examples illustrating how physiological circuits guiding cellular collective behavior impart computational properties to the agential material that serves as substrate for the evolutionary process. I then explore the ways in which the collective intelligence of cells during morphogenesis affect evolution, providing a new perspective on the evolutionary search process. This key feature of the physiological software of life helps explain the remarkable speed and robustness of biological evolution, and sheds new light on the relationship between genomes and functional anatomical phenotypes.

## Introduction

**Fig. 1:**
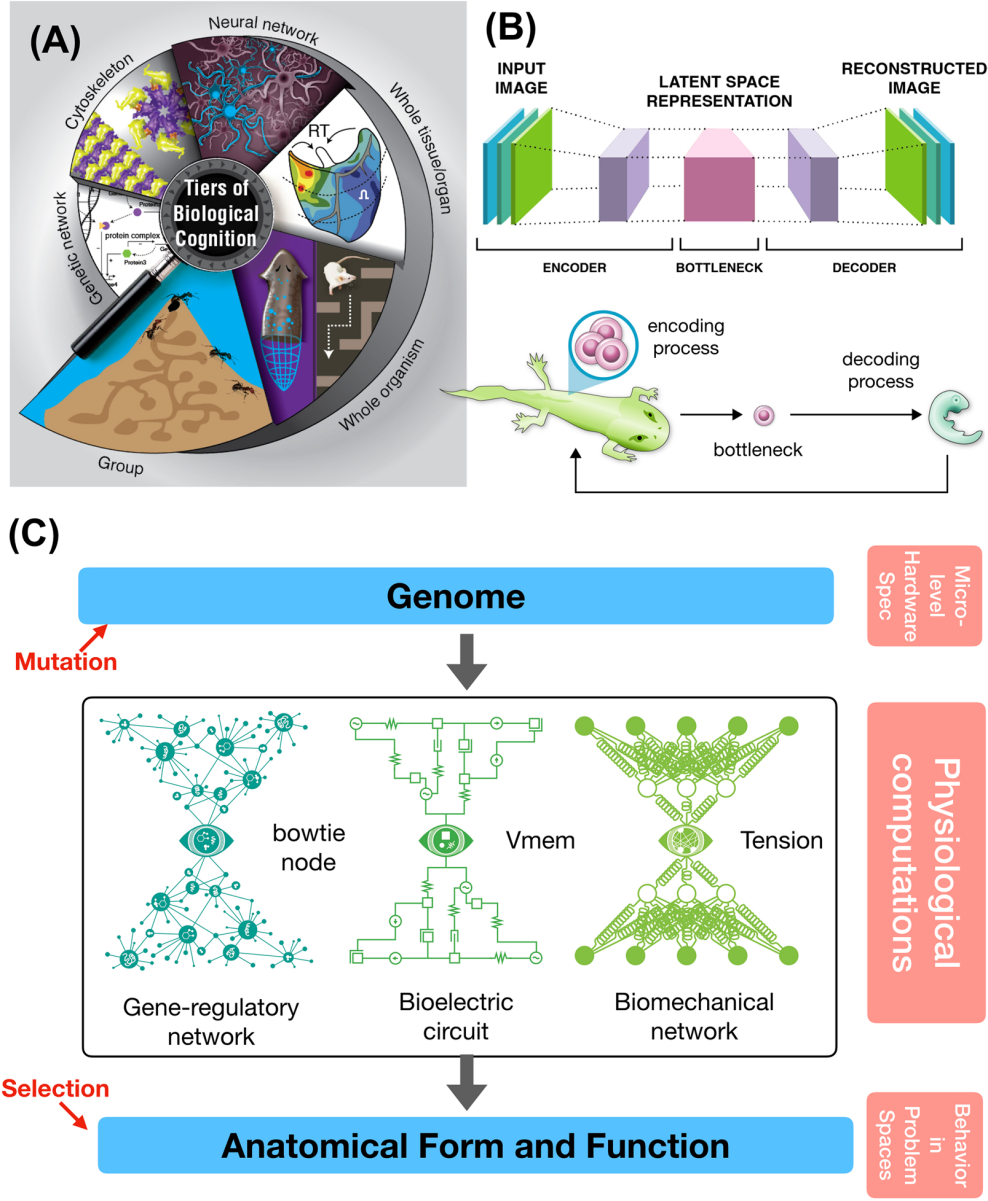
Multiscale architecture of evolution and morphogenesis. **A** Biological systems exhibit complexity at every scale of organization, from molecular networks to whole colonies of organisms. Crucially, these are not just structural levels, but functional: each level’s components are problem-solving agents which navigate physiological, transcriptional, anatomical, behavioral, and other spaces. **B** Many organisms go through a bottleneck that forces a compression of form and function into a generative encoding which does not specify the final structure, but rather specifies a process which creates that form. This is used in machine learning as the “autoencoder” architecture (top sub-panel), which compresses data into a compact representation and then decompresses it to result in an output that is similar at large scale, but not identical in the lowest-level details. The process of reproduction via an egg stage (bottom sub-panel) has the same architecture, except that evolution not only learns the encoding, but also simultaneously encodes the structure of the developmental machinery—the decoder itself. **C** The evolutionary process has not only a genome (the target of mutation) and the anatomical phenotype (the target of selection), but also a critically important layer between them: the morphogenetic physiology that guides embryogenesis, maturation, metamorphosis, remodeling, regeneration, and suppression of cancer and aging. The circuits that implement the computational features of this layer themselves have a bottleneck (or “bow-tie”) architecture, exploiting biochemical, bioelectrical, and biomechanical modalities. These use higher-level “virtual governor” [275, 295] nodes such as calcium patterns, resting potential, or tension—powerful dynamical control points that are complex functions of the underlying molecular details and do not map 1:1 with any gene or gene product. Images in all panels by Jeremy Guay of Peregrine Creative; Panel A used with permission from [14]

The basic workhorse of evolutionary theory is the cycle between the genotype (the target of mutations) and the phenotype (that which selection acts upon). While many models and analyses focus on these key elements, another is often neglected: the physiological processes that underlie morphogenesis. This is the control layer that sits between the genomically specified cellular hardware (proteins) and the form and function that selection sees: anatomy and behavior (Fig. 1). In effect, the behavior of cellular collectives in morphogenesis is the software of the system—the functional outcomes of the molecular machines encoded by genomic information [1]. This is relevant not only for embryogenesis, which converts compressed genomic information into a rich emergent set of large-scale structures, but also for regeneration, metamorphosis, remodeling, and other processes which establish and modify growth and form. Much work has addressed the evolution of developmental mechanisms, the evolvability of specific architectures, and the emergent complexity of epigenesis [2–6]. Moreover, recent work has begun to emphasize the active, cybernetic, problem-solving capacities of this process beyond feedforward emergence [7–11] and explore ways in which evolution increases the functional intelligence of cellular collectives [12–14]. Here, I focus on the complementary side of the evolution–intelligence feedback loop. This is fundamentally distinct from earlier efforts in the adaptationist/selectionist paradigms, and emphasizes problem-solving, unconventional embodied agency, and creativity that are specifically *not* due to adaptation. I first overview the data that illustrate the functional competencies of morphogenesis, casting multicellular growth and form as the behavior of a collective intelligence. I then focus on a fascinating knowledge gap: what implications do the computational properties of the biological software layer have for the evolutionary process itself?

## From genotype to phenotype in development: implications of an indirect pathway

**Fig. 2:**
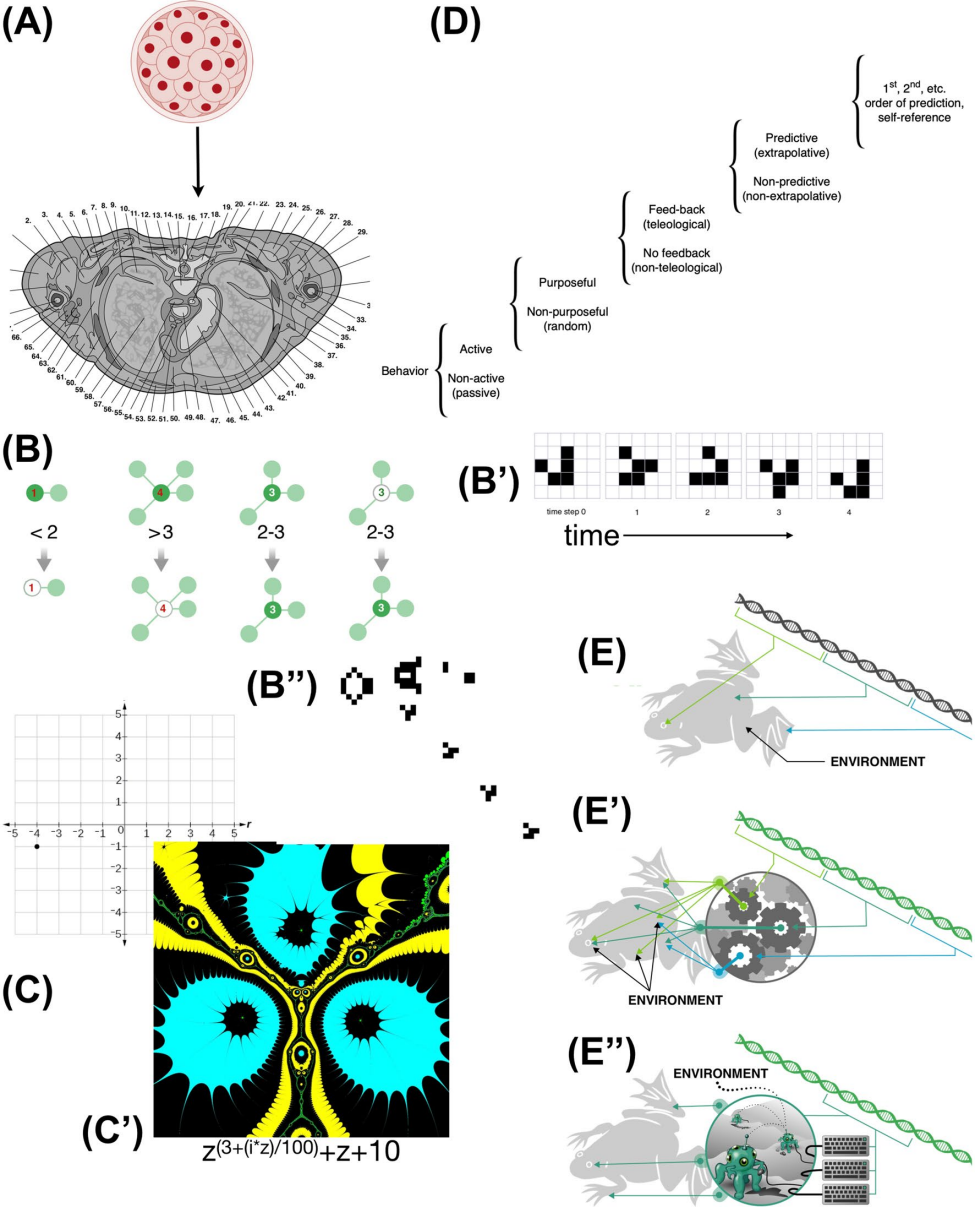
Emergence and genetic encoding architectures. **A** The incredibly complex anatomical structure of an organism (shown here is a cross section through a human torso) is produced by the blastomere descendants of a single fertilized egg cell and its genome, which directly specifies protein sequences (some of the cellular hardware components), not large-scale geometrical features or functionality (nor aspects such as cytoskeletal structure or membrane composition, both of which are inherited parallel to the DNA [206]). **B** One way to achieve complexity via a compressed encoding is through “emergence”. Here are shown four simple rules of Conway’s “Game of Life” that specify the states of a specific cell at each time point based on the state of its neighborhood [296]. Such cellular automata produce patterns that, to a large-scale observer, reveal movement patterns (B’) such as ‘gliders’, and even structures (B”) that continuously produce these virtual moving dynamical patterns (entities), despite the fact that the rules are extremely simple and do not say anything about global patterns or their properties. **C** In the problem space of a complex coordinate plane (2-dimensional grid of positional information), a very simple, local policy can be defined for determining the state of every cell at a position in the grid: a color can be assigned based on how well a root-finding method, such as Halley’s method performs (a simple algorithm [297]). This gives rise to very complex biological-looking forms (C’) complete with multiscale structure and subtle left–right asymmetry. However, all of these kinds of emergent complex systems have a fundamental problem: they are irreversible. What changes could be made in the rules of (B) or the short complex number formula that encodes the image in (C’), to make a glider that moves at a different angle or a pattern that had a different number of blobs and spacing of “hairs” on them, respectively? Such open-loop (purely feed-forward) schemes do not facilitate control interventions (e.g., regenerative medicine) and limit evolution to a simple search of the micro-space of generative rules which is very rugged because the final features are an extremely complex, tangled result of the elements’ following of those rules. **D** A different architecture is suggested by considering that the subunits may be themselves complex components that implement feedback—cybernetic goal-directedness in which energy is used to optimize specific higher-level metrics. The level of control of the subunits that build the organism, as well as of the organism itself (and any swarm of which it may be a part), can span many different competency levels on a scale such as proposed by [99]. **E** Simple picture of genes being responsible for specific features of the organism (which, had it been true, would have made the goals of genomic editing straightforward) has been supplanted with one in which each genetically encoded piece of hardware ends up contributing to many different components of the whole [248] (E’), which raises the inverse problem described above. In contrast, thinking of the genetics as specifying the hardware of computational agents that have competency to solve specific problems (e.g., build an appropriate structure despite changes in cell number, or starting position) offers a much more direct architecture. Closed-loop (homeostatic) agents enable behavior-shaping signals that alter setpoints, not just micromanage hardware components, allowing for a much more linear, tractable, and robust control policy that facilitates evolution as well as biomedical control. Images in panels A, B, E by Jeremy Guay of Peregrine Creative. Panel D used with permission from [99]

One key property of developmental morphogenesis is its emergent nature, in which the relationship between genotype and phenotype is highly indirect. It has long been clear that genomes do not directly code for anatomies; instead, DNA encodes for proteins—the nano-level hardware made available to each cell. The behavior of cells, in a “social” context of multicellularity [15, 16], is what gives rise to functional anatomies (Fig. 2). Cellular behaviors include proliferation, migration, differentiation, shape change, and apoptosis, operating in parallel over millions or billions of cells that are signaling to each other via chemical, electrical, and mechanical modalities—coordinating directly, at long range [17], or by using their microenvironment as a stigmergic scratchpad. This generative process is critical not only during embryonic development, but also in maturation, metamorphosis, regeneration, and suppression of cancer and aging—establishing and maintaining order across multiple scales [18, 19].

A major implication of this architecture is that it is irreversible—while it is straightforward to watch (or potentially to simulate) how biology follows local rules of chemistry and physics and thus to discover what anatomy emerges from a given genome, the inverse problem [20] is in general unsolvable: determining which protein sequences must be encoded to produce an arbitrary, desired large-scale anatomical form. This irreversibility of the recursive, highly emergent process of morphogenesis is what limits full-scale Lamarckism: the difficulty is not how to penetrate Weismann’s barrier and edit the genome in light of somatic experience—mechanisms exist for this [21–23]. Rather, it is *how to know what to change* in a genome to produce a desired feature based on physiological events (e.g., a longer neck). This is a direct consequence of the fact that while the central dogma could, mechanistically, be reversed, the output of the DNA- > RNA- > protein cycle is not anatomy, so reversing it does not solve the problem of going from *anatomical features* back to DNA. This is why we cannot predict the anatomies and morphogenetic capabilities of chimeras made of different cell types [24], despite having genomic information for both. For example, while we have the genomes for frog and axolotl species, one of which makes embryonic legs, we cannot predict if a *frogolotl* (chimeric 50/50 mix) would have legs, and if so, whether those legs will consist only of axolotl cells or both, or whether they will be regenerative like adult axolotl legs. This is because this is fundamentally a question of collective decision-making, which is still poorly understood. In fact, we cannot even predict a single-species anatomy from a genome without first comparing it to a genome whose anatomical outcome we already know. This also sets a ceiling on the biomedical applications of technologies such as CRISPR and other forms of gene therapy: without being able to determine what we have to change to get a specific system-level outcome, our ability to control genomic information to solve complex injuries and disease states will be highly limited. Workers in the field of AI and machine learning are well familiar with this issue as “credit assignment”. However, as will be argued below, this limitation is actually a hugely important intelligence ratchet in biology, because it forces evolution to increase and exploit the competency of its material and the subsequent top-down control (biological innovation via behavior shaping).

The indirect nature and complexity of morphogenesis raises questions as to how evolutionary exploration can give rise to coherent organisms: the search space seems extremely rugged and hard to navigate using the hill-climbing algorithms we associate with evolution. This has given rise to a number of proposals [1, 25–36] to expand the neodarwinian synthesis in various ways. Here, I take a different direction, focusing on one specific aspect of morphogenesis which may provide a critical missing piece in our understanding of evolution: the affordances supplied by the plasticity and problem-solving capabilities of the cells which form the substrate of morphogenesis. Metazoan cells have numerous adaptive behaviors because they derive from ancestral unicellular organisms that needed a full range of behavioral capabilities to survive. Thus, the evolution of metazoan anatomy operates not on a passive material, but on an agential one [37]. I argue that what evolution is really searching is not the enormous space of all possible local rules, but instead the space of behavior-shaping signals by which cells hack each other’s functionality, and that the collective intelligence [38, 39] of cellular swarms has major implications for the rate and course of evolution.

## Insights from collective intelligence and behavioral science: morphogenesis as the work of computationally competent modules

New developments in the science of collective intelligence, and classic concepts from behavior science, are beginning to provide a crucial new lens on morphogenesis that complements the current view of patterning as a complex dynamical system (such as a cellular automaton) with open-loop emergent complexity. This stance emphasizes plasticity and computational competencies for problem-solving in diverse spaces—aspects which need to be dealt with using ideas from cybernetics [8, 9, 40], as well as physics. William James defined intelligence as the ability to reach the same goal by different means [41]. The emphasis is not just the well-known importance of modularity [2], but also the critical cybernetic properties of biological modules and the ways these enable reliability and task delegation (i.e., problem decomposition) across scales in vivo. Together, these features connect readily to concepts such as re-programmability, hacking, predictability, and multiscale polycomputation [42, 43]. This has massive implications for evolution, which are described below.

It is critical to emphasize from the outset that this research program does not support non-naturalistic explanations for evolution, does not make use of exotic influences on mutation such as quantum effects [44, 45], and does not require any goal-directedness in the evolutionary process at the large scale. Instead, it builds on progress in connectionist machine learning [34, 35, 46–50], basal cognition [12, 51–55], and an extension of neuroscience to developmental bioelectricity [56–58] to reveal how the collective intelligence of cells serves as an affordance for evolution. In that, it is complementary to current efforts to understand how network and other generic mathematical and physical properties affect the evolutionary process [59–68]. It should be noted that the below discussion is specifically focused on the traditional view of evolution, avoiding the fascinating and critical roles of epigenotype, epimutations, and other mechanisms that enrich the dynamics of inheritance. My goal here is to examine the major impacts that cellular intelligence has on even a mainstream view of the Modern Synthesis. Thus, I avoid the interesting (and widely debated) aspects of epigenetic inheritance [28, 69–73], which will be incorporated in future work, to focus attention on a number of unconventional hypotheses in the context of an uncontroversial view of evolution.

## Developmental plasticity and evolution today

The implications of developmental architectures for evolution have begun to be explored via the understanding of phenotypic plasticity [74–78] and the way that behavioral agency of whole organisms shapes how evolution explores the latent space of phenotypes [77–82]. For example, the changes in skeletal features, behaviors, and physiology of fish and amphibia in novel stressful environments [83, 84] have been suggested to bias evolution and strongly complement traditional mutational variation as a source of change [77, 78, 85]. However, the crucial aspect is not simply that the complexity of development enables changes by real-time events, and that the genome does not uniquely specify one possible outcome. More than that, a view of morphogenesis as simply a recursive, massively parallel process limits bottom-up control via genetic editing in biomedical engineering for the same reason it limits the efficiency of evolutionary search across micro-level properties of a passive material. It is proposed that for the same reasons that bioengineers are now turning to top-down control policies and interventions [37, 58], evolution has discovered an architecture that exploits its unique substrate: cells and tissues with specific competencies, behaviors, and input-driven decision-making computations.

It is proposed that the plasticity of development and its implications for evolution cannot be optimally captured by an exclusive dynamical systems perspective in which different outcomes simply occur as the result of different environmental inputs working their way through a mechanical process. It has been suggested that morphogenetic mechanisms exist on a continuum of problem-solving capacities that exists across scales within the biosphere [54, 77, 81]. Evolution can thus be seen as not only shaped by organism-level learning and intelligent behavior [27], but also by physiological and anatomical level intelligence [55]. This perspective links the plasticity of the morphogenetic process with the field of basal cognition, which seeks early evolutionary origins of classical intelligence and problem-solving in behavioral science by looking at lower level examples of adaptive behaviors that rely on information processing. Cells and their components have many competencies for adaptive behavior when navigating physiological, metabolic, and transcriptional spaces [86]. Examples include the ability of gene-regulatory networks and chemical pathways to learn from experience [87–89], for transcriptional machinery to rapidly adjust to compensate for entirely novel physiological stressors [90], and for unicellular slime molds to explore their environment at a distance and make decisions using memory and comparison of alternatives [91–95].

## Intelligence and evolution: the impact of competency at new scales

The impact of intelligence on evolution has been studied [36, 96]. However, it has largely focused on intelligence of the organism (classical behavior in 3D space), and is only now beginning to be extended to higher levels such as the intelligence of swarms [97, 98] and entire ecosystems [49]. The framework discussed herein extends the study of the relationship of intelligence and evolution in several basic ways. It extends the notion of intelligence to sub-organismal scales, casting morphogenesis as the result of collective intelligence at the molecular cell, tissue, and organ levels. It operates within a gradualist [14] perspective on intelligence and goal-directedness [57, 99], which are both used here in a naturalistic, cybernetic, engineering sense of varied degrees of competent problem-solving in diverse spaces by unconventional agents (i.e., not restricted to higher-level cognitive capacities in brainy animals). Thus, it emphasizes morphogenesis as a computational process, not merely the emergent outcome of highly parallel local rules that could be dealt with by established complexity theory tools. Moreover, it expands the concept of intelligent behavior across a key invariant: effective navigation of diverse problem spaces, which includes problem-solving in physiological, metabolic, transcriptional, and anatomical spaces [12, 13, 86]. With these expansions in hand, it becomes possible to see how evolution pivoted some of the same strategies across these domains, and to then explore the implications that this multiscale competency architecture has for the evolutionary process itself.

Several open problems are addressed below. What are some of the features of morphogenesis that enable evolution to search a very difficult space (with pleiotropy, degeneracy, redundancy, etc.)? How can evolution be so rapid and effective at using mutation to address opportunities of form and function? How do we explain the ubiquitous presence of novel capabilities in living things for which no direct selection forces are plausible? Analysis of morphogenesis as the behavior of a collective intelligence of cells leads to the following key proposals. First is that the space which evolution actually searches is not only the space of microstates of the genome, but also a much more tractable space of behavior-shaping signals: evolution exploits cellular intelligence as a highly exploitable affordance. Second is that by using a variational autoencoder-like architecture of compression into a generative genome (Fig. 1), evolution is freed from over-training on past conditions and pushed to evolve general-purpose problem-solving machines which inevitably display robustness, plasticity, and adaptive success in entirely novel circumstances. Interestingly, these ideas are more familiar concepts in neuroscience; consistently with the recent advances in basal cognition [100–104], these concepts apply long before complex brains appear. My hope is that the following analyses extend an active research program on these ideas in regenerative medicine [58, 105–109] and synthetic bioengineering [37, 101, 110–113] to also impact evolutionary biology and engineering of artificial systems via evolutionary algorithms [96, 114–119].

## Scaling up from cellular competency to collective intelligence

Basal cellular competencies did not disappear during the transition to multicellularity: instead, they scaled up as a collective intelligence to operate in larger and more complex problem spaces [13]. Models of mechanisms by which single-cell competencies potentiate problem-solving capacities in larger and different problem spaces include gap junctional merging of intracellular milieus and other forms of tissue-level signaling (e.g., stress propagation). This promotes sharing of chemical engrams, which also help to wipe the individuality of cells, and enlarges the memory, predictive capacity, and sensory/effector radius of cellular networks, enabling them to pursue larger goal states in spaces like anatomical morphospace [14].

The key feature of scaling up individuality (whether evolved or engineered) is for higher levels of control to get their components to do things they do not do when operating as individual units [46, 120]. This can be discovered by extracting the parts of organisms and examining their behaviors in new contexts [121]. A cell-level example is that fragments of keratocytes move in a specific direction in an electric field, but whole keratocytes (collections of such fragments) actually move in the opposite direction [122]. A morphogenetic and behavioral example is seen in Xenobots [123, 124]. Frog embryo skin cells, in vivo, form a two-dimensional, passive layer on the outside of the animal that protects it from pathogens. However, when liberated from the instructive influences of the other cells, frog epithelial cells instead form a Xenobot—a functional, self-motile construct with many novel behaviors that are normally suppressed and hidden by the instructive signaling of other cells during development [125]. Human tracheal cells from adult donors also form such biobots, with the remarkable ability to traverse and heal neural wounds [126]. Thus, it is not obvious what the default morphogenetic behaviors and capabilities of cells are, because of the ubiquitous dominating controls of other cells in their environment. In some cases, embryonic capacities (e.g., formation of appendages) are “lost” at maturity (e.g., limbs in adults of many species), but can be activated in non-regenerative contexts by specific stimuli that can be provided by bioengineers in the context of regenerative medicine [127–129].

**Fig. 3:**
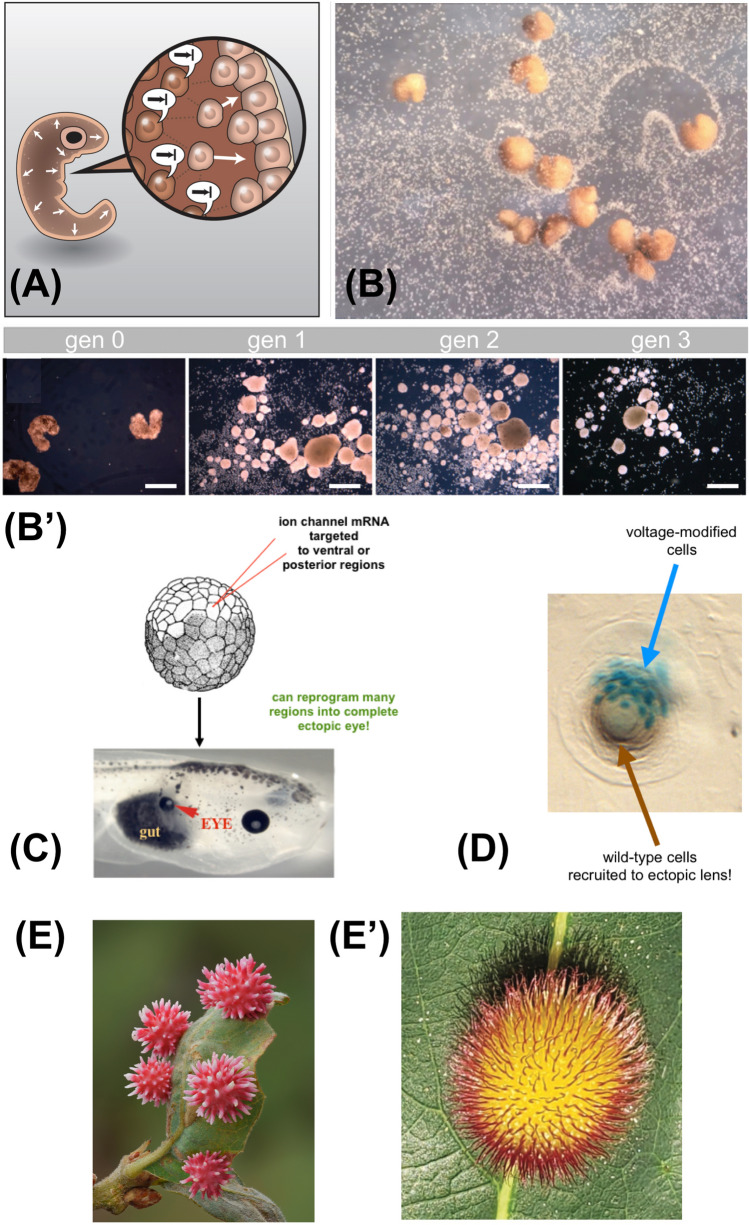
Morphogenetic hacking. **A** Morphogenesis is implemented by cells, which were once organisms themselves. Thus, the process of anatomical homeorhesis [252, 298] is implemented by a set of behavior-shaping signals in which some cells control the behavior of other cells. Image by Jeremy Guay of Peregrine Creative. **B** One example is seen in the behavior of frog skin cells, which produce a passive, two-dimensional outer layer in a normal frog embryo, but when freed from the instructive interactions of other cells become Xenobots—spherical constructs that move autonomously and even exhibit entirely new behaviors such as kinematic self-replication (B’), in which they assemble loose cells into clumps that become the next generation of Xenobots and repeat the cycle in subsequent generations [124, 125]. Panels B, B' by Douglas Blackiston, used with permission from [125]. **C** When bioelectric signals that indicate “build an eye here” are reproduced in other locations of a frog embryo via potassium channel modulation [152], a complete eye can be formed even out of gut cells without needing to micromanage the construction of a complex organ—the modular trigger induces a morphogenetic process that can reliably take place in novel environments. Panel C used with permission from [148]. **D** The process is more than just triggers of master-regulator signals. When an insufficient number of cells is modulated (indicated in blue beta-galactosidase tracer), they recruit their normal (unmodified) neighbors to complete the task of building an ectopic lens of the right shape and size. This ability to complete a particular action in morphogenetic space by controlling the behaviors of other cells in a context-sensitive, dynamical manner is a key feature of controllability and reliability—it is how the problem-solving capacities of cells, not just ability to execute the same hardwired local rules, facilitate adaptive robustness in novel circumstances. Panel D courtesy of Sherry Aw, used with permission from [37]. **E** The controllability can be exploited even in cross-kingdom interactions (not just by bioengineers), in which plant cells are induced to form morphologically highly novel structures known as galls by signals from fungi or insect embryos: the novel structures, which differ greatly from the default, are an example of natural hacking of morphogenetic machinery (E = urchin gall on a scrub oak, by Timothy Boomer at WildMacro.com; E’ = hedgehog gall, by Andrew Deans)

These and other examples indicate that biological components are themselves, to varied degrees, autonomous, but are controllable by signaling from other cells [37]. The dynamic of using simple signals that take advantage of the recipients’ complex, reliable repertoire generalizes to the concept of *hacking*, which is applicable at multiple scales and in many contexts (Fig. 3), ranging from chemical signals in cellular induction to colony-scale behavioral phenomena driven by acoustic signals [130]. The crucial focus in this concept is on the role of an agent that takes advantage of affordances in its own way, not necessarily in the ways “intended” by an engineer, or by evolutionarily-prior functions. Past discussions of relevant phenomena include developmental niche construction [131, 132], cancer cells reprogramming normal neighbors [133], host-altered behavior due to parasites that exploit neurotransmitter mechanisms [134] (and the reverse—the host evolving machinery to manipulate the parasite [135]), zombie ants controlled by fungi [136], the explore/exploit strategies of young mammals [137], and many others. An amazing example of morphogenetic hacking is the formation of galls, where signals from a parasite force the leaf cells away from their normal flat, green tissue phenotype and into building spiky, three-dimensional colorful forms [138].

Many such phenomena work because of the agency of the material being hacked. This includes viral infections since viruses have very limited capabilities and rely on the cell for all of its physiology; the use of toxins to manipulate hosts [139]; and the phenomenon of kinematic self-replication by Xenobots. These motile synthetic organisms make copies of themselves in an evolutionarily novel manner: when provided with loose cells, the biobots corral them into piles which self-compact and thus spontaneously form the next generation of biobots which go on to repeat the cycle [125]; this only works because the material itself is competent to form a viable Xenobot when rearranged (Fig. 3). At higher levels of organization, one can see classic organism-level communication and signaling as examples of hacking the more agential elements of one’s environment (i.e., the conspecifics, predators, and prey contained in it).

Thus, the concept of hacking extends well beyond the typical strategies used for traditional engineering with inert materials, or computational matter (as in, the computer science notion of hacking [140]), to systems with significant agency: hacking is conceptually linked to *behavior shaping*. In this perspective, everything in biology is a hacker, reaping rewards of efficient manipulation of its environment (and its internal components) using the appropriate tools (from direct chemical effects to subtle signals meant to be interpreted by complex agents): cells, parasites, viruses, transformed cells defecting from the morphogenetic goals of a tissue (cancer), tissues and organs during morphogenetic induction and competition (even within the same body [141, 142]), whole organisms, and swarms. Evolution can thus search the space of behavior-changing signals, exploiting the complex, agential nature of the cells which are its substrate as a hugely powerful set of affordances [143].

## Bioelectric control: a mechanism for scaling cell behaviors to large-scale morphogenetic goals

**Fig. 4:**
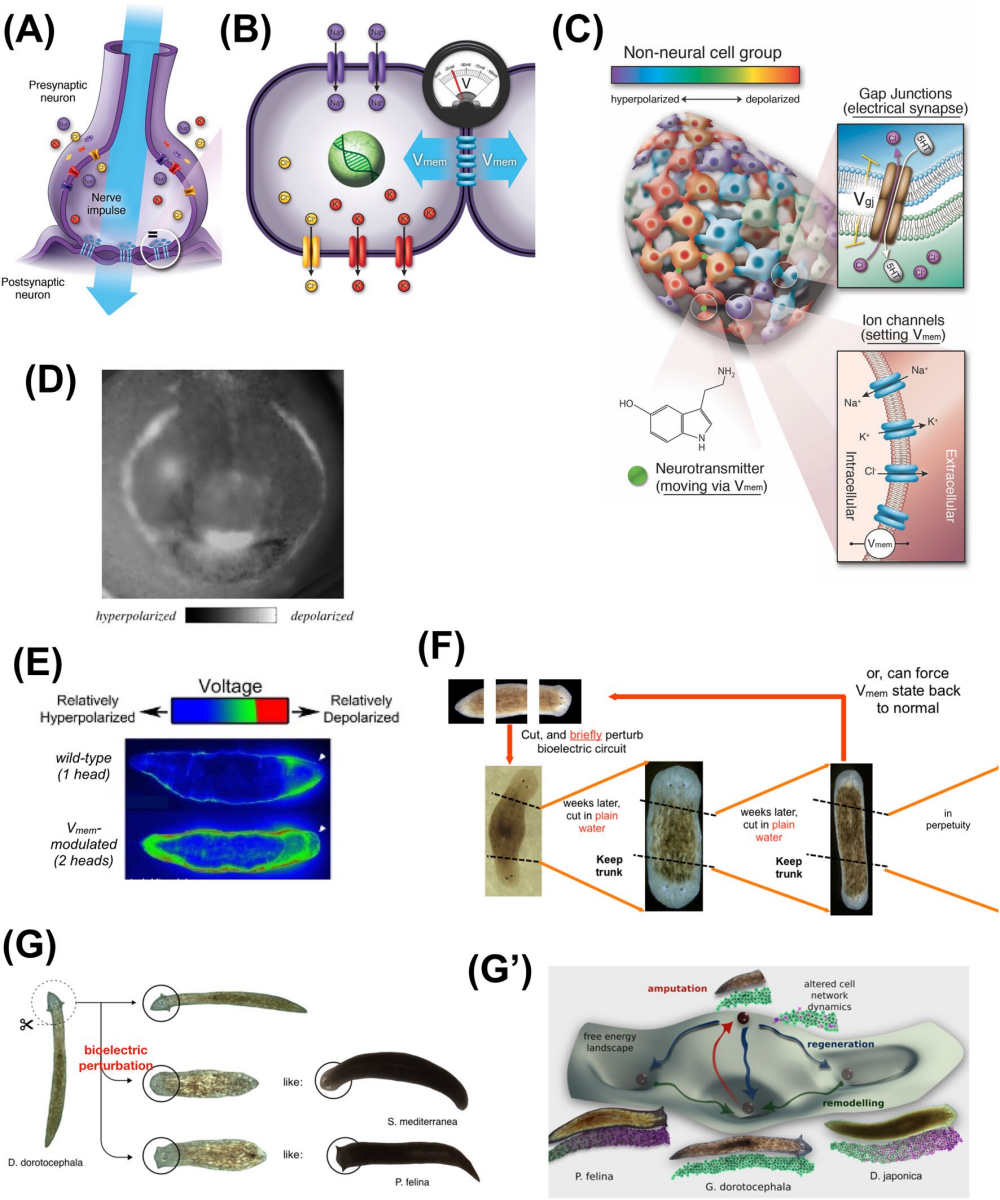
Bioelectric machinery underlying morphogenetic control. **A** The computational architecture of the nervous system relies on a network of cells, which set their resting potential via ion channels in their membrane, and communicates that state via controllable electrical synapses known as gap junctions [299]. **B** This machinery is ancient, with all body cells having resting potential across their membrane (V_mem_) mediated by the same ion channels and most cells making gap junctional connections to form bioelectrical networks [188]. **C** This interface can be manipulated in any tissue in the same way as neuroscientists probe brain tissues: using molecular reagents to control connectivity (via opening and closing gap junctions) or directly setting voltage states by opening and closing ion channels (using drugs or optogenetic light stimulation). Also, the downstream messengers (neurotransmitters) operating in many tissues can be controlled directly [300, 301]. **D** An example of imaging of the bioelectrical prepatterns that define morphogenetic setpoints is the “electric face” revealed by signals from a voltage-sensitive fluorescent dye that indicates the future positions of the gene expressions, and subsequent anatomical structures, of the early frog embryo anterior ectoderm. This pattern is instructive because if it is shifted artificially and induces the predictable changes in subsequent development. Note the simplicity of the encoding—the bioelectrical states represent high-level order (elements of the target morphology for regulative development), such as the location of an “eye” or “mouth”, not the state of gene expression or individual stem cell fate. **E** The bioelectric representation of the target morphology guiding the cellular collective can work as a *counterfactual* memory, as in the example of the cryptic planaria: animals with normal one-headed anatomy and gene expression, but a two-headed target morphology representation in the bioelectric circuit (shown here using the voltage dye imaging), which causes them to build two-headed animals if cut into fragments [193]. **F** The bioelectric prepattern is a true *memory* because, despite the wild-type genetic sequence, fragments from two-headed animals continue to generate two-headed animals in perpetuity (with no more treatments) [195]—this is an example of the software layer that is reprogrammable and enabled by the genetically specified ion channel hardware, which does not need to change to be shifted to an entirely different large-scale target morphology. **G** Not only can head number be reprogrammed by a brief physiological stimulus to the morphogenetic agent, but also head *shape*: an animal with a triangular head shape can be shifted toward morphologies (including brain shape and stem cell distribution) [197, 198] of other species with round or flat heads. This enables the cellular collective to explore attractors in morphospace (G’), using the exact same genome, which eventually could become canalized into genetically distinct species. Panels in A, B, C are courtesy of Jeremy Guay of Peregrine Creative; A, B used with permission from [188]. Panel D is used with permission from [150]. Panel E is used with permission from [106]. Panels F, G are used with permission from [107]. Panel G’, by Alexis Pietak, is used with permission from [197]

One especially powerful interface. which is exploited by evolution to accomplish morphogenesis, is the bioelectric layer of control (Fig. 4). Cells perform numerous computations in controlling their behavior via the voltage dynamics of ion channels and pumps [107]. But evolution discovered very early on, around the time of bacterial biofilms [144], that electric networks are also extremely convenient ways to integrate and process information across space and time, and to scale the functions of individual computing units towards a large-scale task (the same reason that brains, and computers, use this modality [145]). Several examples from the field of developmental bioelectricity illustrate this point.

For example, in frog embryos, a set of cells known as “instructor cells” [146, 147] normally suppress melanocytes’ native behavior via serotonergic signals. In the absence of these signals, the melanocytes exhibit an over-proliferative, hyper-invasive phenotype strongly resembling melanoma [148]. Even more interestingly, the bioelectric signaling scales independent decision-making of the cells toward a collective outcome. When the control system is perturbed, stochastic outcomes (e.g., 70% conversion) result, but only *at the level of the organism—*each animal’s melanocytes are either all normal or all converted, at some stochastic rate: they toss a coin, but all the cells are tossing the same coin. It required a machine learning algorithm to analyze this control system and discover an intervention that breaks the concordance and enables individual melanocytes within the same body to make independent decisions [149]. This bioelectric signaling, which involves serotonergic control, parallels what nervous systems do to bind individual cell behaviors, even stochastic ones, toward a system-level collective behavior.

The vertebrate face [150, 151] is patterned by a set of bioelectric gradients that regionalize gene expression to determine the locations of eyes, mouth, and other components. Reproducing these patterns elsewhere in the body via misexpression of specific ion channels enables the induction of whole ectopic organs, such as eyes [152] on the gut or tail of a tadpole (thus unlocking novel capabilities of body regions, since only the anterior neurectoderm is competent to make eyes via signals from the chemical master regulator Pax6). Remarkably, when only a small number of cells are injected, they recruit their normal, unperturbed neighbors to participate in the process to make a normal-sized lens (Fig. 3). Thus, in response to the bioengineer’s instructions to “make an eye”, the prompted group of cells not only activates all of the required downstream molecular events (which are not directly specified by the simple, trigger-like stimulus supplied), but also determine how many additional cells are needed for large-scale morphogenetic setpoints such as size control and signal to activate them as well. This ability to scale recruitment of elements to task size is similar to that seen in collective intelligences such as colonies [153, 154].

Crucially, this is not just modularity [3] (“do the same thing again, in another place”), but takes advantage of cellular swarms’ competency to adjust dynamically to circumstances as needed, because the modules are not structural or hardwired. The cybernetic lens is appropriate here (and fruitful) because cell groups demonstrate diverse degrees of ability to accomplish a defined set of outcomes despite changing circumstances and can be manipulated top-down. This system for integrating the behavior of cells and tissues offers many advantages for phenotypic richness, but like any programmable interface is amenable to exploitation. For example, the bioelectric system for control of head shape and location in planaria [155, 156] can readily be hijacked by microbes that can control the number and structure of their host’s heads [157]. It is straightforward for evolution to also exploit these modular competencies: to reproduce an eye in another location, evolution does not need to reproduce all the underlying mechanisms.

## Morphogenetic control as a collective intelligence

“Intelligence: the ability to reach the same ends by different means.”

― William James

The current paradigm for understanding morphogenesis focuses largely on ideas from emergence and complexity theory. It is clear that some local rules, executed repeatedly and in parallel by large numbers of cells, can result in an emergently complex structure (Fig. 2). This is true, and we now have good mathematical formalisms for understanding emergent complexity. However, this is a limiting paradigm because it is entirely feed-forward, or open loop: causality is thought to move in only one direction (from the molecular details upward) [158, 159], and thus all modifications have to be made at the level of the rules. Because the inverse problem is not generally solvable, this view locks in a model of evolution limited to searching the genotype space (which may be extremely rugged, due to its nonlinear relationship to the phenotype space). It also limits workers in bioengineering and regenerative medicine to exclusively targeting the molecular hardware in hopes of improving system-level outcomes (which in turn results in the difficulties with drug discovery [160]).

Fortunately, there are now tools available to begin to think in different ways about morphogenetic control, which facilitate new research and make possible a new roadmap for discovery of intervention policies, both by bioengineers and by the evolutionary process. The first is cybernetics [99, 161]: by emphasizing the information-processing capacities of multiscale components of living systems (with all of the attendant implications of circular control, multiscale causality, etc.), it becomes possible to recognize the reliability of morphogenesis as a consequence of the goal-directedness of underlying processes. It is essential to abandon the traditional scientific teleophobia [162] because cybernetics and control theory now provide a mature, naturalistic, quantitative science and engineering approach to mechanisms with goals. Goals do not require magic, they require a specific causal structure [163, 164], which is ubiquitous and not restricted to complex brains. The critical importance of the cybernetic worldview for developmental biology has been recognized already [7–9, 165–167]. Additional tools are provided by advances in information theory which have now rigorously shown that higher levels of organization can have causal power and *drive* lower-level events in the only sense that matters: by serving as the most effective control points at which to make changes in the system [168–172]. This is as relevant for regenerative medicine as it is for evolution, and is becoming a very useful lens through which to relate to biological systems.

The crucial transition is recognizing that homeostatic cycles, the atoms of cybernetic systems, are not merely feedback loops (which are widely accepted as ubiquitous in biology), but are the first rung on a spectrum of *intelligence* [99]. Intelligence is used here in William James’ definition, not limited to advanced metacognition in primates. The field of basal cognition [53, 54, 173] seeks to unravel the evolutionary origins of the brain’s remarkable trick—unifying the activity of millions of cells (neurons) toward a common purpose in behavioral space. By examining the transitional forms that smoothly continue all the way down to microbial molecular networks, a wider understanding of diverse intelligence has had to be forged [14, 55]. From this, the view of the brain as a collective intelligence has been enlarged to understand the morphogenetic transformations of the body as a collective intelligence of cellular swarms, which solve problems in other spaces [39, 86]. Thus, the robustness of development is not of first order (do the same thing reliably each time), but of higher degree (achieve the same target morphology, by various means, despite various perturbations).

**Fig. 5:**
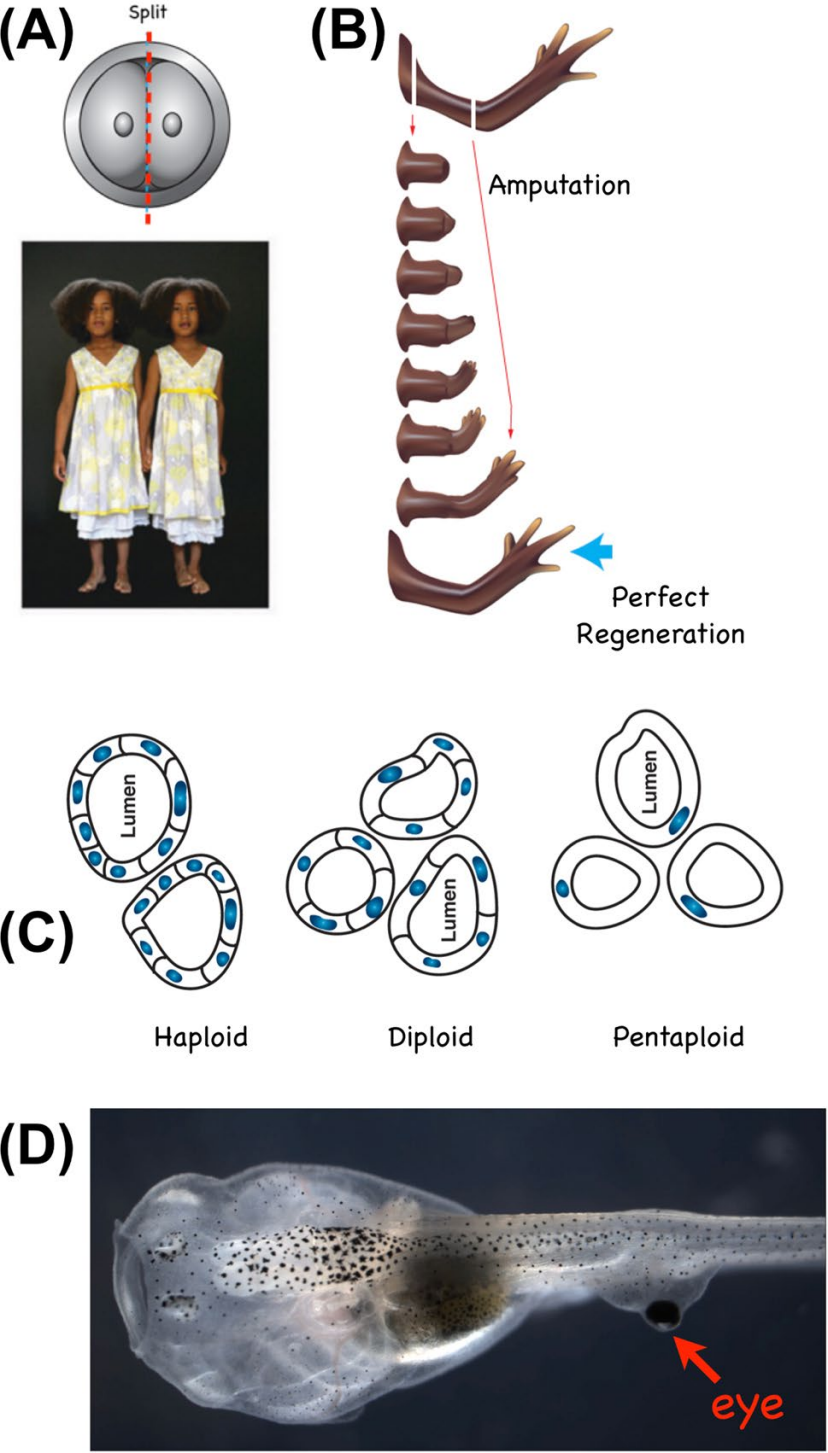
Examples of morphogenetic and functional competency. **A** Many embryos, including human, can be split in half at early stages and give rise to multiple normal whole bodies (monozygotic twins), making up for very drastic damage to nevertheless achieve the correct target morphology. **B** The same is true of regeneration, in which for example a salamander limb can be amputated at any level, and the cells will rapidly rebuild exactly what was missing, stopping when the correct target morphology has been achieved. **C** A remarkable example of top-down control occurs when newt cells are made to be larger than normal, by forcing polyploidy [181, 184]. The same overall kidney tubule structure and diameter results, despite the incorrect details at the lower level (excess genetic material, and wrong size of building components), as the cells adjust their number to the new size—this happens in real time (developmental timescale) not requiring evolutionary changes in the genome. Most amazingly, a different underlying molecular pathway is triggered (cytoskeletal bending, vs. cell–cell communication) when the cells are made so large that the only way to build the structure is for one single cell to wrap around itself, leaving a topological lumen. **D** The plasticity is not only structural, but also functional. When eye primordium cells are transplanted to the tail, they not only make a correct eye despite abnormal environment, but the resulting animals (which can be made to lack primary eyes) can efficiently see out of these eyes, which do not connect directly to the brain [176]. This does not require evolutionary adaptation to the new sensory system architecture, illustrating the amazing ability of body components to make up for unexpected changes induced by mutation or other means: a property that has huge implications for the evolutionary process. Panel A photo by Oudeschool via Wikimedia Commons. Panels B, C by Jeremy Guay of Peregrine Creative. Panel D by Douglas Blackiston

Taking evolutionary continuity seriously and applying concepts from cybernetics reveals an important invariant: navigation in problem spaces as a common set of capacities that range across a continuum (Fig. 5) of competency. Different degrees of intelligence are thus cached out in engineering terms as abilities to overcome barriers in the problem space, in a wide range of diverse inorganic, organic, and neural-level systems. This generic formulation enables recognition, prediction, and manipulation of systems across a continuum that covers all the intermediate levels of sophistication that lie between two magnets’ ability to get together and Romeo and Juliet’s ability to get together [14, 86]. But, these competencies cannot be definitively ascertained from observations of the default course of morphogenesis, which obscures the ability of living tissue to handle novelty (of both, external environment and internal composition) and lulls the observer into a limited expectation that genomes code only for specific outcomes and no more. A very rich set of examples belie this misconception and instead support a view of morphogenesis as a goal-directed, homeodynamic process [11, 174].

The most obvious examples are seen in regulative development and regeneration [57, 175], where cells work to implement and maintain a large-scale form (target morphology) despite surgical, genetic, and physiological sources of defects. But it goes much further than that. Tadpoles, in which the native eyes are prevented from forming and an ectopic eye is instead placed on the tail, can see and perform well in visual behavioral training [176], even though the ectopic eye connects to the spinal cord (or just to peripheral tissue) rather than to the brain—this radical change to the sensory–motor architecture does not require generations of adaptation to produce successful behavior. Tails grafted to the flanks of amphibians slowly turn into limbs [177]—a structure more appropriate to the large-scale target morphology (even though, for example, the local environment for the tail-tip cells is perfectly correct); and craniofacial structures that start out in odd positions are often corrected [178]. Frog skin cells, forbidden access to the normal reproductive and other capacities of an entire embryo, nevertheless create a functional proto-organism that makes copies of itself through kinematic replication, a mode unknown within the natural tree of life [125]. Perhaps the most remarkable example of this is Slijper’s goat [179], in which the effort of trying to walk upright (due to lack of forelimbs) drove, in one generation, many of the anatomical and physiological changes usually thought to require long periods of evolutionary adaptation to bipedalism.

These kinds of abilities to achieve target morphology are not unique to damage-repairing programs that evolved specifically to handle injury. They occur at all scales of organization, suggesting they may be a general capacity arising from our microbial ancestors’ need to respond to changes in their environments and in their own genomes. For example, mice with mutated semaphorin proteins still produce (through a novel path) correct connectivity of migrating thalamocortical connections [180]. Even more radically, animals artificially made with extra copies of their entire genomes, which produces huge cells, still exhibit normal size [181–183]. In the kidney system of polyploid newts, the normal cell:cell communication that creates a tubule from eight to ten cells working together is swapped for a single enormous cell that wraps around itself to create the same final outcome [184]. This is a good example of downward causation, because in the service of a large-scale anatomical goal, different molecular mechanisms (cell–cell communication vs. cytoskeletal bending) are deployed as needed for an unexpected change (a drastic increase in the copy number of all genes). The ability to handle, without periods of evolutionary change, an unexpected alteration in something as basic as one’s own genome and cell size echoes the theme that has been seen in the study of behavioral learning and evolution [36, 185]: degrees of behavioral intelligence can produce adaptive outcomes on timescales that are much faster than evolutionary search for hardwired phenotypes for each problem, and on landscapes whose ruggedness is too high for the basic evolutionary search process.

**Fig. 6:**
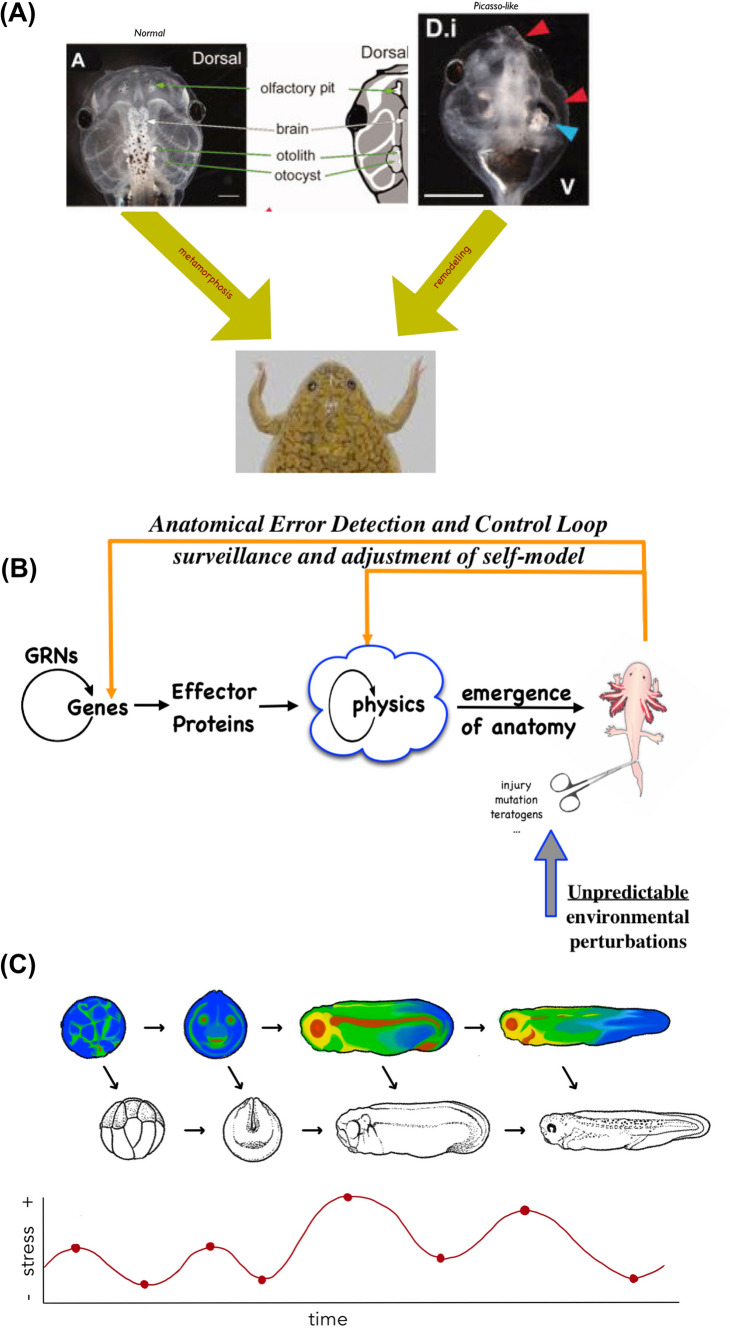
Pattern homeostatic properties of the morphogenetic collective agent. **A** The normal process of rearrangements of the tadpole face to make a frog face is not hardwired to specific movements. Embryos in which the craniofacial structures are scrambled also make largely normal frog [178], which shows that these components form an error-minimization system for the correct target morphology and has the competency to reach it even when the initial positions are incorrect. **B** This kind of ability to navigate anatomical morphospace with policies beyond hard-coded actions (see the scale of possible competency levels in Fig. 2D) adds an important component to the mainstream open-loop scheme of traditional gene regulatory networks driving interactions, which result in emergent complexity at the anatomical level. The view of the cellular collective as a cybernetic system, which represents goal states for morphogenesis (see their direct visualization in Fig. 4D,E), enables specific research programs to detect, decode, and re-write target morphology information without needing to rewire the genetic hardware—an attractive prospect for regenerative medicine [57, 58, 303]. **C** The concept of morphological homeostasis during regenerative repair could be extended naturally to the broader concept of morphological homeorhesis, in which developmental progression is a collection of regenerative repairs [57]: each stage is in effect a “birth defect” from the perspective of the subsequent stage and is “repaired” by regulative development which seeks to minimize error (i.e., system-level stress) relative to the bioelectric target morphology. This perspective hypothesizes that the bioelectric target pattern changes more rapidly than the transcriptional and anatomical patterns, pulling them along by stress-minimizing loops that have been scaled up to act on larger metrics than single-cell stress states [304]. Maturation and adulthood result when the bioelectric and biochemical prepatterns stop changing and the anatomy essentially catches up and future changes are small-scale maintenance and resistance to aging and carcinogenic defections. Panel A taken with permission from [178], and courtesy of Erin Switzer. Panel B by Jeremy Guay of Peregrine Creative. Panel C by Brenda B. de Groot

Seeing development, metamorphosis, regeneration, and cancer suppression all as manifestations of an anatomical homeostasis loop (Fig. 6) raises the obvious question of how and where the setpoint (target of homeostatic error minimization) is stored. Having been predicted as far back as the 1940s [186, 187], it has recently been found and manipulated using the same concepts and tools that have allowed neuroscientists to manipulate goal-regulating behaviors in living brains.

## Bioelectric networks as reprogrammable interface to morphogenetic capabilities

**Fig. 7:**
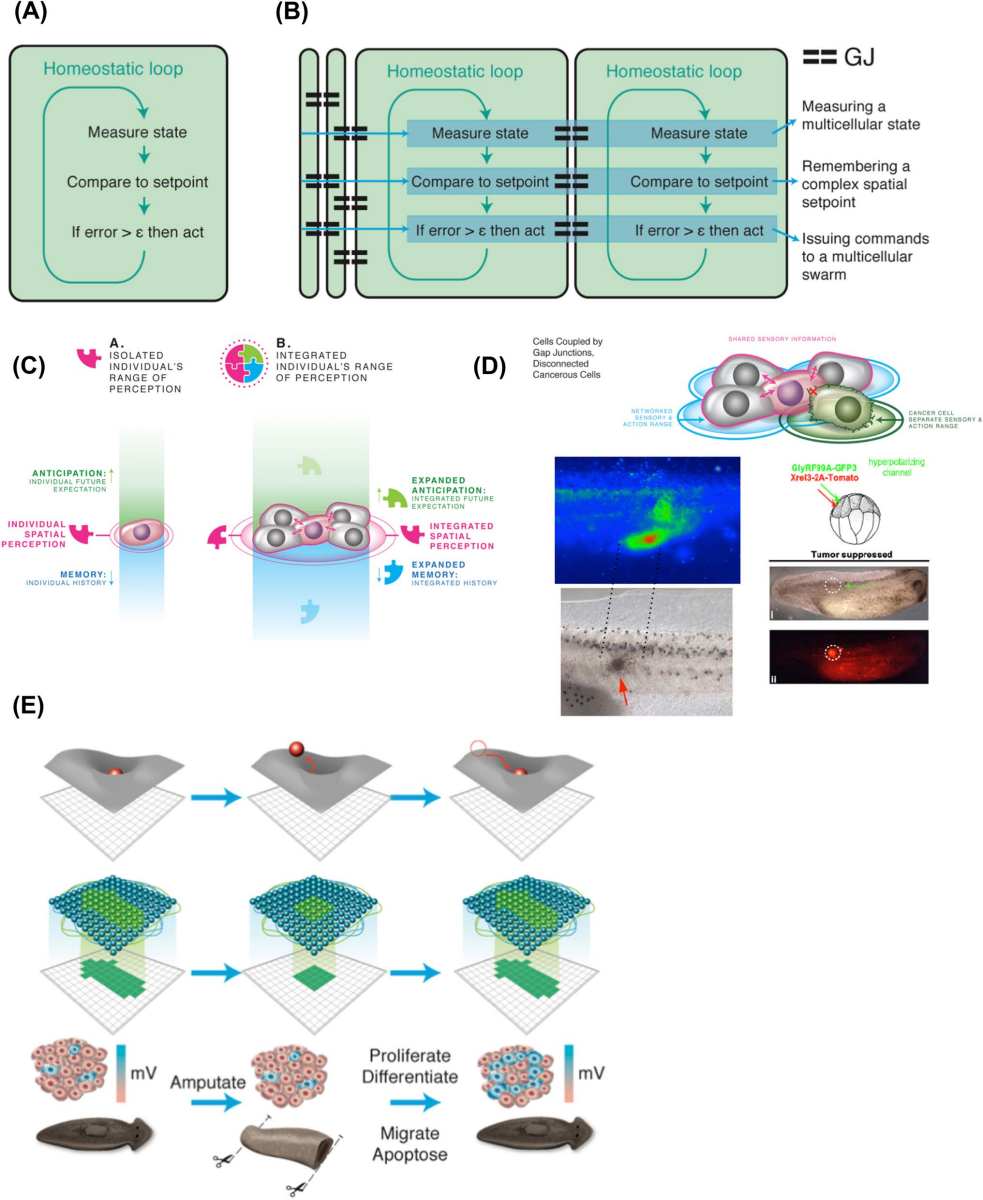
Scaling of the morphogenetic agent. **A** The components of a complex morphogenetic system can be thought of as cells with minimal computational abilities, for example the homeostatic capacity to keep cell-level parameters (e.g., pH) within certain levels. **B** For the collective intelligence of morphogenesis to competently navigate anatomical morphospace, in addition to cell-level physiological space, these subunits have to connect into networks that computationally store much bigger goal states (e.g., rough representations of coordinates in anatomical space, or target morphology shapes). It is hypothesized here that this occurs via gap junctional dynamics, but the details of the scaling of setpoint states are an active area of current research [295, 305–310]. Of course, biochemical and biomechanical modalities are also very likely to be involved in the scaling of competencies from subcellular to multicellular problem spaces. **C** In effect, the scaling enables cellular collectives to enlarge their “cognitive light cone”—the spatio-temporal scale of the target states they are able to pursue, increasing their memories of the past, ability to predict future states from past experience, and the spatial extent over which they can measure current states [13]. **D** Current work is ongoing on the open problem of linking these cybernetic perspectives to the biophysics of the state space of the electrical circuit of a multicellular tissue, and to central ideas in connectionist machine learning which show how a network (excitable medium) can store large-scale patterns that are robust to perturbation and can be recovered after damage [57, 58, 270]. **E** The consilience of computer science approaches to cognition (connectionist models of memories in networks), dynamical systems views of systems navigating landscapes, and developmental bioelectricity provide an emergent unification of the notions of memory and recall in morphogenesis and behavior. Panels in C,D,E by Jeremy Guay of Peregrine Creative. Embryo panels in D used with permission from [311]

Evolution broadly exploits bioelectric networks for the same reason they underlie cognition in the brain and computation in our information technology: they have some very attractive properties with which to build generic learning and problem-solving systems [58]. All cells participate in electric networks, which allows them to compute and increase the size and complexity of simple homeostats (Fig. 7). Neurons speed-optimized the process and took advantage of direct long-range connections, but other than that, many of the tricks that brains use to handle novelty are in fact prevalent across the body, long predating classical neuroelectricity on both evolutionary and ontogenic timescales [145]. Many aspects of connectionist models of neural networks’ computational capabilities apply to developmental events [34, 46, 58, 120]; for example, regeneration is a kind of pattern completion in anatomical space, and stochastic phenotypes arising from the same genome can be studied as a kind of perceptual bistability [106].

Bioelectric networks mitigate the inverse problem by serving as the “hidden layers” of a control network linking genetics to anatomy (Fig. 1). The relationships of ion channel genes to the bioelectric pattern, and bioelectric pattern to its resulting anatomy, are each easier to invert than the direct genotype–anatomy link, so they help to decompose the control problem into two easier problems. This not only helps natural evolution, but also helps engineers design and evolve complex proto-cognitive circuits, and increases control capacities for workers in regenerative medicine. Bioelectric networks provide modularity (triggers of complex subroutines, such a simple voltage state that triggers the “build an eye” or “build a leg” subroutine [188])—a known component of evolvability [3, 189]. They also provide an important kind of coarse graining, since voltage is a high-order parameter over ion channel gene and protein microstates, and individual ion concentrations: electrogenic proteins can be swapped out as needed (e.g., V-ATPase complex in the tadpole tail can be phenocopied by a yeast proton pump with no sequence or structural homology, because it produces the correct physiological signal), and everything still works as long as the bioelectric state is correct [190]. Like in the central nervous system, bioelectric interfaces that control tissue- and organ-level structure [107] are very attractive control nodes. It is no surprise that viruses, whose genomes are under significant pressure to remain minimal and concise, devote some of that precious space to ion channel genes [191, 192].

Bioelectric circuits also provide another crucial competency: memory, which is a specific instance of their more general capacity for software-level reprogrammability. As in the brain, bioelectric circuits can store information in a way that is readily changeable in the lifetime of the animal and enable the exact same (genomically specified) hardware to have different modes of activity depending on prior experience. For example, in planaria, the bioelectric circuit that controls the number of heads can be targeted with a brief (2-day) physiological intervention that re-sets the state of the circuit to specify “two heads” instead of the default one-headed bioelectric pattern memory. In this case, the exact same hardware (genetically wild-type planarian cells) can harbor multiple representations of the bioelectrically encoded target morphology that guides the cellular collectives during regeneration [193–195]. The question of what determines the number and shape of heads in the planarian is subtle, paralleling the question of what determines behavior. It is genetics in the sense that genomes specify the ion channels needed to form the circuit, but it is not genetics in the sense that the actual outcome is controlled by experience (in physiological and behavioral space, respectively). Evolution could make use of this in precisely the same way as it exploits classical learning (brain bioelectric dynamics), via genetic assimilation and Baldwin effects [29, 196] that can later canalize adaptive outcomes of morphogenetic and physiological plasticity. Indeed, it has been shown that existing planarian species’ head shapes are readily recapitulated by a genetically wild-type animal experiencing changes to its bioelectric circuit during regeneration—100–150 million years of evolutionary distance in morphospace can be crossed in a few days because of the dynamics of bioelectric pattern memories [197, 198]. If proven advantageous, this could eventually be transferred into the genome by ion channel mutations that produce the same bioelectric circuit-driven developmental behavior (assimilation of physiological plasticity into the hardware [199]).

## Multiple modalities for computation and control across scales

As ideal as bioelectric networks are for the plasticity and control dynamics that potentiate evolutionary change, they do not have a monopoly on this role (Fig. 1). Other mechanisms, operating at other scales, provide the same benefits [60]. For example, the richness of cytoskeletal information processing [200–202] enables cortical inheritance in unicellular organisms: there, as in planaria, DNA is not the only source of morphogenetic information. Changes made to unicellular form can persist across generations [203–205]; for example, surgical rotation of a small patch of the cell surface creates a stable line of animals in which a portion of the ciliary beat pushes food out of their mouths—a condition which their perfectly wild-type genome cannot correct. The cytoskeleton serves as a plastic information and control medium stretching (parallel to DNA) all the way back to our last common universal ancestor [206]. The availability of a rapid, stable but re-writable, internal scratchpad is an important component of any system that is to exhibit ability to deal with novelty and to navigate problem spaces in a flexible, dynamic fashion.

Another subcellular component with interesting proto-intelligent competencies is the class of pathways, which includes both protein interaction and gene-regulatory networks. These have been shown to underlie anticipation and probabilistic inference [207–209], six different kinds of memory [87–89, 210, 211], and other capacities that evolution can exploit in the control of individual cell behavior and thus organ-level morphogenesis. An example of transcriptional problem-solving is seen when planaria are cultured in barium [90], a non-specific potassium channel blocker. At first, their heads degenerate (due to the many cells that require K^+^ flux), but they rapidly re-grow new heads that are completely barium insensitive. Transcriptional analysis reveals that these tissues up- and down-regulate just a handful of genes to get back to physiological and anatomical setpoints despite the inability to regulate potassium. The remarkable thing is that barium blockade is evolutionarily novel—planaria very likely have never encountered the exotic stressor of barium during their evolutionary history, and there have been no selection pressures to develop specific responses to it. Their ability to navigate physiological and transcriptional spaces to solve an entirely new problem is an example of subcellular competencies for novel scenarios (and perhaps, include the ability of generalization from scenarios that they did encounter, such as epilepsy-induced excitotoxicity). Finally, the use of chromatin as a computational resource has been suggested to be an important driver in evolutionary transitions [212, 213].

Increased understanding of the capabilities of cells and their components dovetails with efforts in modern robotics in which morphological computation (via biomechanics [214, 215] and bioelectrics [216]) is heavily exploited—the emphasis is not on developing a specific controller that does all the hard computational work for a fixed body, but in evolutionary approaches that exploit bodies’ innate competencies [217]. By being easy to control, the body does much of the hard work of the evolved controller [96, 218, 219]. This is an important dynamic for understanding how multiscale intelligence shapes evolution: much as we examined *existing* pathways to find a perspective from which they appear as trainable systems amenable for example to Pavlovian conditioning without any structural change [88, 89], evolution can implement behavior-shaping signals that are equivalent to a control perspective on its substrate that recognizes its agential affordances and seeks to exploit its inherent competencies, not only micromanage the underlying details. Indeed, evolved pathway networks show much more capacity for learning than random ones [88, 89], serving as an example of evolutionary enrichment of such properties.

The evolutionary origins of these competencies are an active area of study: the field of basal cognition seeks to understand how evolution gives rise to increasing competencies in navigating diverse problem spaces [14]. Here, however, the focus is on the second half of the loop (Fig. 1): how do these problem-solving competencies affect the evolutionary process itself? The roles of basal agency in evolution are now beginning to be discussed [77, 220, 221]. Models are being formulated for understanding how each layer of the multiscale competency architecture (MCA) of life deforms the option space for the layers above and below it (Fig. 7). The above-mentioned examples of induced lens cells recruiting others, and of artificially large cells using a different molecular mechanism to complete tubulogenesis, demonstrate behavior shaping and top-down control. We can exploit this in engineering contexts, but it is certain that evolution discovered the power of exploiting these affordances as well. Next, we consider specific ways in which this dynamic drives an evolutionary ratchet for problem-solving capacities and makes sure that the results of evolution are often not specific solutions to specific environmental niches, but rather general purpose problem-solving machines, whose capacities cannot be guessed, or inferred from the invariant, default course of development.

## Beginner’s mind: self-organization on the fly as the origin of multiscale competency

“If your mind is empty, it is always ready for anything, it is open to everything. In the beginner's mind there are many possibilities, but in the expert's mind there are few.”

― Shunryu Suzuki

The standard view of evolution emphasizes two things. The first is the cumulative nature of the process—each change is made in the context of prior events and thus is strongly dependent on history. Concepts like ontogenic recapitulation of phylogenetic events stress the fact that developmental mechanisms must operate with whatever components and signals had been established before (were adaptive within the prior historical context of that species). This is true, but there is a complementary aspect, because of the ubiquitous unexpected scenarios that every embryo has to face: environmental changes, genetic mutations, physiological stressors, parasites, and numerous other challenges that cannot be planned for in advance. Thus, biological systems evolve under pressure to remain flexible enough to accomplish coherent morphogenesis despite a wide range of perturbations.

This means that the most successful, robust embryogenic and regenerative processes must not assume prior states strongly. The above-mentioned examples show how little embryos can take for granted. They cannot assume how many cells they have (ability to regulate pattern despite available cell number [222, 223]), the genetic complement and size of those cells (polyploid newts [181, 184]), the starting position of specific structures (unscrambling frog faces [178]), or that earlier molecular signaling steps were completed correctly (repair of mis-steps in the left–right asymmetry pathway [224]). Evolution uses a mix of pre-specified features and “wildcard” phenotypes which are handled by real-time problem-solving [36]. When evolution is seen as a long-term learning process [35, 48–50, 225], it becomes clear that it does not over-train on its priors: past experience shapes development, but for most creatures (with exceptions like the mosaic *C. elegans*), the mechanisms that survive are ones that are not hardwired to specific expectations but are ready to operate despite a wide range of noise and both internal and external uncertainty [35].

This concept can be encapsulated with the term Play the Hand you’re Dealt (PHD): successful developmental systems are primed to operate with whatever internal and external conditions they are faced as they come into the world (within some broad, but limited, range). PHD is why biological systems are highly interoperable with exotic synthetic materials [226], why huge mechanistic diversity exists within individuals of an evolutionary type [227], and why viable chimeras can be made at every level (from mixing DNA to cell/tissue grafts, to parabiosis [24], to hybrots in which brains can be used to drive novel robotic bodies or live entirely in virtual worlds [174]). This extends to behavior and neuroscience: we are *all* “brains in a vat”, and our neural systems can readily adapt to sensory–motor augmentation with engineered devices or novel sense organs [228, 229] because of the fundamental plasticity that requires each being to learn about its own form and function in real time. Functional behavior from tail-eyes [176] or third arms [230] or video game embodiment all works because developmental processes are primed to figure out their sensors, effectors, and architecture in real time [231–234].

Genomes not only do not encode large-scale features, but they also do not even exclusively encode machinery that always makes the same large-scale features: they specify cellular hardware that solves problems at multiple scales. This includes dynamically setting boundaries between the self and external world [108], self-modeling [235, 236], and exploiting available problem spaces [86]. In addition to homeodynamic loops, biological systems exploit affordances of the laws of physics and computation to benefit from the learning capacity of network architectures [34, 46, 49, 88, 89], the informational scaling provided by strong cell:cell coupling [13], and the coordinating capacity of competition [141, 142]. All of this could result from the multiscale competency architecture, in which systems from molecular pathways to whole animals (though not being units of selection directly) solve local problems in an active manner with diverse degrees of competency [14]. This allows evolution to readily pivot through problem spaces (Fig. 7), using the same general computational tricks to handle navigation in metabolic, physiological, transcriptional, morphological, behavioral, and linguistic spaces [86]. Like the principal components describing the activity of artificial neural architectures, these spaces are often not obvious to us as external observers. For example, bacteria that can take actions such as physical movement, or transcriptional changes of enzyme expression, to address dissatisfaction with local sugar levels, operate in a hybrid problem space with effectors that operate in what we call gene expression space and 3D space, respectively.

Successful creatures must operate with a kind of “Beginner’s Mind” with respect to the collective intelligence of morphogenesis. There is an interesting parallel here with neuroscience, in which behavior is a combination of past history and on-the-fly decision-making. Much as each organism must interpret its physical engrams in real time to select actions, morphogenesis is a dynamic process that acts by interpreting the genetic material with which it must work. For example, Xenobot cells interpret the same *Xenopus laevis* genome in a way that results in kinematic self-replication and other behaviors, while standard frog embryos exhibit the more common frog-like interpretation. Like in learning, the ideal memory is not a recording of microstates (which often cannot be reused in other contexts), but a compressed, generative representation of what and how to optimize—second-order policies that are usable even when details change.

Morphogenetic problem-solving capacity must be seen at a level higher than that of cells. A focus on cellular plasticity and cell-cycle checkpoints leads to predictions that animals with easy access to undifferentiated cells (e.g., regenerative ones) should be highly prone to cancer; in fact, the true situation is the opposite [237]. Animals with robust morphogenetic control due to high levels of plasticity are resistant to changes of circumstance (injury) at many scales: they regenerate after loss of limbs and organs (degradation of body-scale information), they suppress cancer readily [238] (degradation of tissue-scale information) and they resist aging (degradation of cell-level information).

The best examples of this counter-intuitive dynamic are planaria, which not only recover their entire bodies from even small fragments, but are very cancer resistant and apparently ageless. This raises a crucial puzzle for the traditional view of genomes as specifying form and function. Why does this extremely “long-lived” animal (greatly outshining animals like humans or elephants, in which long-term cancer suppression is commonly touted) avoid cancer despite consisting of about one-third its cell number as stem cells? Moreover, because planaria often reproduce by fission, any mutation that does not kill the stem cell is propagated into the next generation and expanded, resulting in animals that are mixoploid chimeras with an extremely messy genome [239]. How does the animal with the messiest genome have the best morphological control?

## An evolutionary intelligence ratchet

A possible answer to this puzzle merges the above concepts of evolution producing versatile problem-solving machinery. Of course, the problem-solving competencies are themselves produced by genetically encoded hardware, suggesting the view of two kinds of genomic information: that which directly specifies phenotypes (e.g., sequence of protein enzymes, or structural genome) and that which specifies a problem-solving competency (second-order computational capacities). This in turn provides a potential explanation for planaria. In hardwired individuals, in which the genome directly specifies various features of the phenotype, the fitness reveals a lot of information about which genomes are most adaptive: selection can see the quality of the structural genome. However, in individuals in which the morphogenetic process has competencies (such as adjusting the position of a misplaced mouth, establishing functional vision with a tail-eye, or adjusting physiology in light of a stressor such as barium), selection has a hard time picking the best genomes because some of its successful instances are in fact not due to a great structural genome, but to a competency of the parts to adjust. Because cells can navigate morphological and physiological spaces, fitness carries less information about the structural genome.

It has been suggested that when evolution cannot make efficient gains by optimizing the hardwired components, the remaining targets for optimization are the competency mechanisms themselves [240]. This starts a feedback loop, because each gain in competency makes it even harder to judge the structural genome, which exacerbates the drive toward improving competencies—a ratchet for multiscale intelligence that can readily be seen in computational models of the process [240]. Planaria, salamanders (which regenerate, but are not immortal), and mammals all represent different degrees of how far this ratchet has operated in their lineages, because other forces oppose it (e.g., complexity drain [241]). This phenomenon is familiar for example in human evolution, in which case evolutionary pressure for the largest muscles has been lifted, because the most successful reproducers are ones with high computational capacity which use manipulation (e.g., tools, language, and medicine) to increase their reproductive success, making it hard for fitness to select for the ones that are physically the most robust.

This model explains a number of very puzzling observations, beyond the fact that the messiest genomes (400 + million years of somatic inheritance) have the most robust anatomies—a striking disconnect between genomic and morphological stability. For example, it predicts the confirmed fact that there are no known mutant planarian strains with abnormal morphologies (the way there are for fruit flies, mice, etc.) and explains why the research community has had such a hard time (despite decades of efforts) generating transgenic planaria. In this lineage, the ratchet has run all the way forward, optimizing mechanisms to create a functional body (almost) no matter what the genome looks like: all of the effort has gone into polishing a set of algorithms that produce a functional anatomy *despite expected noise* in the components, which then makes it very difficult to create change by targeting the genetic level. In fact, the only known permanent strains of planaria with an abnormal anatomy are two-headed [195] and destabilized (random 1 vs. 2 head) forms [193], which were not made by genetic change but by manipulating the physiologically implemented software that stores pattern memories and guides planarian tissues in their navigation of physiological and morphological problem spaces. This illustrates the profound link between the course of evolution and the dynamically changing competencies of its substrate material [37]. In the next section, specific implications for the evolutionary process itself are discussed.

## Implications of multiscale competency for the evolutionary process

“It is not the strongest of the species that survives, nor the most intelligent that survives. It is the one that is the most adaptable to change.”

— Charles Darwin

**Fig. 8:**
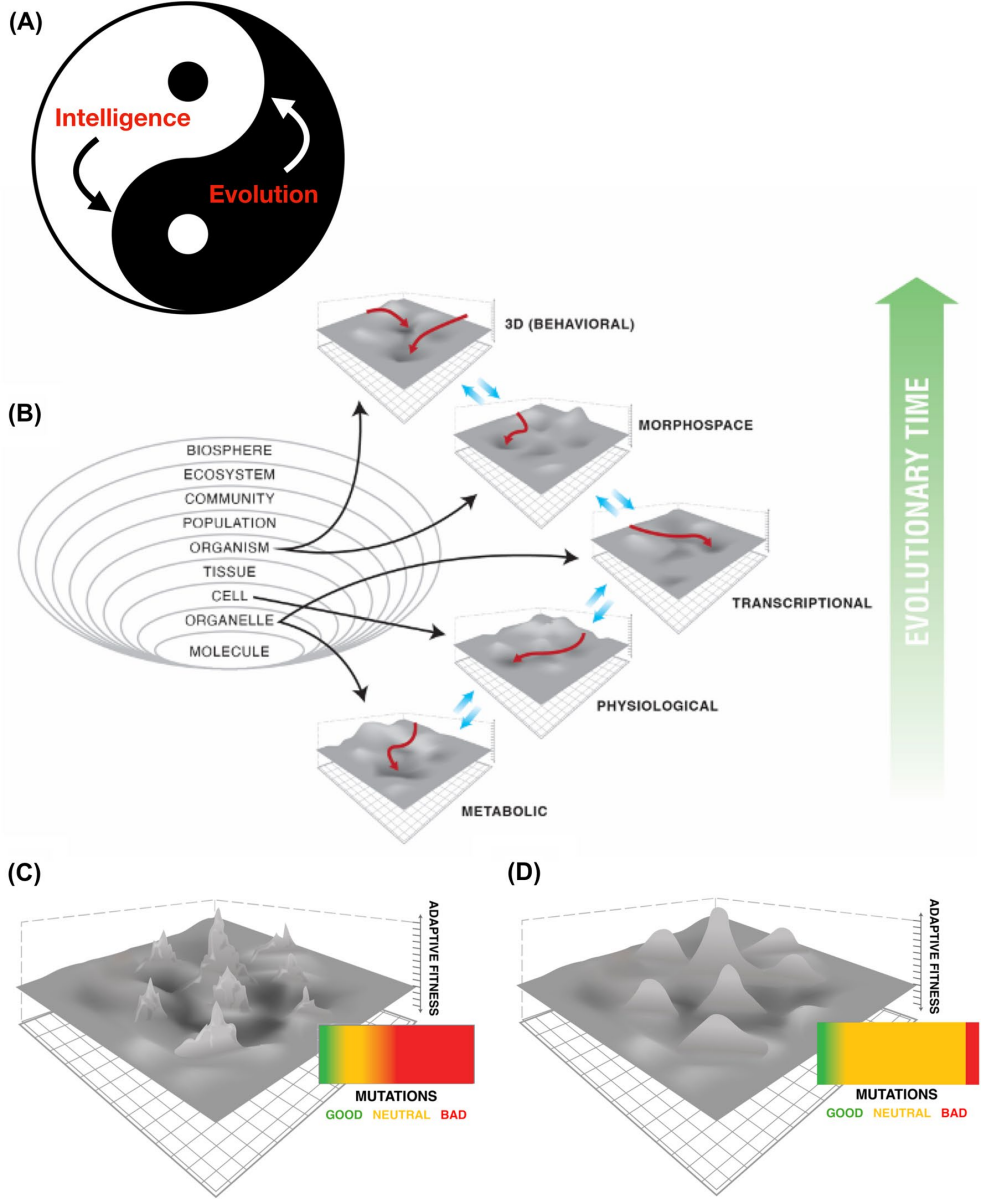
Summary of the evolutionary perspective on morphological competency. **A** It is well appreciated that evolutionary dynamics have scaled intelligence, from the primitive capacities of microbes to the metacognition of modern humans. However, it is suggested here that the process is symmetrical: the intelligence of the components (i.e., problem-solving capacity) potentiates the evolutionary process, forming a ratchet that enhances this capacity while it makes it harder for selection to see the structural genome [240]. **B** It is suggested here that the multiscale competency architecture (the cybernetic nature of each layer) enables evolution to pivot across problem spaces, using the same navigation policies to solve problems in metabolic, physiological, transcriptional, morphogenetic, and 3D spaces (the latter being the traditional space of “behavior”). For example, the (bioelectric) computations of neural networks that control muscles to move the body through 3D space emerged from much earlier usage of those same mechanisms and algorithms to control other cell types [145] to navigate the configuration state of the body through anatomical morphospace despite novel circumstances (examples in Figs. 5,6) [86]. Blue arrows symbolize how evolution scaled up each type of control into a new, larger space. The intelligence-focused lens on the co-developmental process suggests that the competency of the layer between genotype and phenotype (developmental physiology) greatly smooths the rugged (**C**) evolutionary landscape, resulting (**D**) in a faster search and exploration for novel capabilities. The ability of cellular and tissue-level agents to accomplish their tasks in their respective problem spaces despite unpredictable changes enables many mutations that would otherwise have been deleterious to be neutral, as the system adjusts and enables evolution to explore other, potentially positive effects of pleiotropic mutations. Mutation is thus just one of many internal and external perturbations that deviate the body from a target morphology—a ubiquitous stressor that needs to be dealt with on developmental and evolutionary timescales. On this view, regenerative capacity is not a novel solution to external damage, but an ancient, ubiquitous aspect of homeostasis that enables developmental progression, regulative morphogenesis, and also efficient evolutionary search. Panels B, C, D used with permission from Jeremy Guay of Peregrine Creative.

The multiscale competency architecture (MCA) was foreshadowed in the notion of semiautonomous processes and dissociability by Needham, Gould, and others [242–245], but it has not heretofore been sufficiently appreciated. The ubiquitous embedded agency of evolution’s substrate means that the layer between the genotype and selection can make up for a wide range of errors and novel circumstances. In some cases, the correct species-specific target morphology is recreated (e.g., scrambled embryonic frog faces), while in other cases a different functional morphotype emerges (e.g., Xenobots). The same is true in physiological and transcriptional cases (e.g., planarian barium adaptation). These are not merely environment-induced differential developmental outcomes, but the ability to reach specific outcomes despite internal or external perturbations. Beyond the advantages of encapsulated modules and their triggers, the problem-solving capacity of morphogenesis enables the larger system to delegate tasks and rely on them being completed in a wide range of circumstances that do not have to be anticipated and micromanaged. The following are ways in which the MCA potentiates, accelerates, and shapes evolutionary processes (Fig. 8).

1. By providing an agential substrate, MCA speeds the evolutionary search for better solutions in several ways. Active, modular problem-solving capacity enables the following properties for the evolutionary process:
  o *Generalization.* By increasing the number of genotypes that all map to the same functional phenotype, the partially autonomous generative layer helps evolution generalize [35, 50]. When functionality and thus fitness are not tied to a single molecular implementation, significant plasticity and robustness arise via exploration of different ways to achieve the same goal, generating alternatives which might prove to have other advantages. For example, the instructive property of resting potential (V_mem_), distinct from the specific ion levels that underlie it, means that a bioelectrically controlled morphogenetic function can continue unaffected, while evolution experiments with other consequences of swapping out different channel proteins in that circuit.
  p *Reliability.* Modular agential systems can persist and be continuously improved and built upon, because the ability of the parts to achieve specific design specs enables the other parts to functionally trust that they will accomplish their task even when changes occur. This makes it practical to invest energy in complex regulatory systems. MCA facilitates cooperation (which is not guaranteed by mere genetic relatedness [141]) between components and across levels of organization by encapsulating complex behaviors in a simple module with a specific goal. Systems that have a clear, reliable goal state that they can be depended upon to pursue are easier to cooperate with, and thus present a lower barrier for investment in cooperation.
  q *A more tractable search space.* By smoothing the fitness landscape, MCA makes the search process much more efficient, improving evolvability by enabling the search to overcome local maxima. By reducing phenotypic differences between different genotypes, MCA enables selection to explore the consequences of mutations which would otherwise be maladaptive. For example, a mutation which displaces the mouth, but has some other beneficial impact elsewhere, will not result in a dead embryo (and thus failure to exploit the other consequences of that mutation) because the mouth will self-correct [178].
  r *Cryptic variance.* MCA makes many mutations neutral that would be deleterious in a direct genotype → phenotype architecture. Such mutations whose effects are mitigated by developmental regulation are not weeded out; instead, they persist, generating over time a rich pool of diversity that may be phenotypically dormant, but can be exploited in future. Cryptic variation [74, 246] has been proposed to underlie adaptation to previously unencountered conditions or environments.
  s *Functional intermediates.* Goal-directedness (in the cybernetic sense) of developmental modules helps resolve the problem of useful intermediates, because the autonomous problem-solving capacity of the parts pushes partial solutions toward an attractor. In *On the Origin of Species*, Darwin wrote, "If it could be demonstrated that any complex organ existed which could not possibly have been formed by numerous, successive, slight modifications, my theory would absolutely break down." Many scenarios which, in a flat architecture, would result in an in-between state with low fitness, instead, end up in a useful attractor state because of the “do until condition X is met” character of homeostatic and homeodynamic loops.
2. Implications* for the inverse problem.* An intervening developmental control layer can help mitigate the nonlinear relationship between genotype and phenotype at the micro level by providing a more linear relationship between control signals exerted at higher levels of organization and phenotypes.
  o *More linear controls.* It is likely that a layer of cybernetic competency makes the relationship between genotype and phenotype more linear [20, 247]—addressing the difficult inverse problem of selecting which genes must mutate to reach a system-level outcome. This potentially empowers epigenetic mechanisms that go beyond a completely blind search of the genetic space. By encapsulating the chaotic (in the mathematical sense) relationships between changing local rules and emergent outcomes into modules with distinct goals, the system becomes easier to control via a combination of top-down encoded goal states and bottom-up emergence.
    As described above, morphogenetic goal state encodings (such as bioelectric prepatterns) can be re-written despite a wild-type genome. Keeping such goal state encodings functionally orthogonal (independently modifiable) from the machine that executes them is a powerful architecture exploited by computer science. This separation, complementing feed-forward emergence, progressively minimizes the inverse problem at each pair of layers, allowing much more efficient functional architectures.
  p *Facilitating credit assignment between changes and positive outcomes.* Evolution does not have to wait until a solution is found that improves one property while not impairing others—it can exploit the much easier route of testing out properties, while the error-correcting competencies of underlying modules mitigate the fact that most genes have many different roles [248]. MCA helps handle the fact that it is very hard for any optimization process to make progress when each change has many different consequences, by reducing the need for determining individual contributions for each control signal.
3. MCA’s modularity confers both robustness and hackability.
  o *More control at the higher levels, less at the lower.* The advantages of robustness (making some components controllable by other components) result in encapsulation of cybernetic modules which are more controllable in specific ways (goal rewriting, behavior shaping), but less controllable in their details (they resist noise and intervention, and sometimes have novel unpredictable behaviors—a hallmark of the primitive roots of agency). It may be far easier for evolution to exploit complex outcomes by making changes in the setpoint information (e.g., by manipulating ion channel structure) than by trying to micromanage local rules that are many steps away from system-level outcomes. Future work will measure the relative improvement in edit distance [249] between highly fit organisms in bottom-up vs. behavior-shaping evolutionary approaches.
  p *Evolutionary pressure to detect ‘hijacking’.* However, the increased controllability, and thus reprogrammability, of components is also a potential target for exploitation by parasites and various cheaters [250], as it makes it that much easier to control complex outcomes in host or conspecific behavior. This in turn sets up an evolutionary pressure for systems to model their own functionality (causality) to detect when their control circuits are being changed internally or externally. This reinforces the boundaries between self and world (individuation) and may have many consequences for cognitive capacities in more advanced life forms.
4. MCA facilitates evolutionary creativity.
  o *Pivots to novel problem spaces.* Another way evolution can exploit the benefits of the MCA is by changing the *measurement* component of the homeostatic loops, which generates novel functions (and thus jumps across the fitness landscape) by providing new *perspectives*—new ways one component (or the larger system) can control and exploit on an existing mechanism—polycomputation [42]. More broadly, useful computational functions, such as associative memory, can be derived from a gene-regulatory network *without changing the structure of the network* (and thus without any possibility of adversely affecting any dependents). A mechanism that interprets its outcomes in a particular way (maps specific nodes to the functional elements in an associative conditioning paradigm for example, [88, 89]) is readily evolved and modified, and provides a way for evolution to squeeze additional benefits from existing components without the negative consequences for other internal observers (subsystems), thus keeping prior gains. Future work must examine the role of perception (in the active inference sense) and polycomputing in potentiating evolutionary advances, to see how much of the effort of evolutionary optimization is spent tweaking the measurement, setpoint, or actuation machinery of homeostatic loops.
  p *The competency ratchet* described above [240] and the facilitation of complex high-level control loops by the modularity of the test–operate–exit loops of homeostatic mechanisms go beyond simple problem-solving. They also help evolution address novel *opportunities*—open-ended [251] exploration of new spaces and selection of novel problems to solve, not just better ways to solve the same problem. One example of this is the ability of planaria to find transcriptional paths to resolve an entirely novel physiological stressor (barium) [90]. Another is the ability of Xenobots made of wild-type *Xenopus laevis* cells to perform kinematic replication (which, to our knowledge, no other organism does), suggesting that more focus needs to be placed on how allostasis, homeorhesis [252], and autonomization [253] not only guide navigation in an established problem space using developmental motifs (a.k.a., behavioral modules in anatomical morphospace), but also enable pivoting across novel spaces—a hallmark of early agency. Fundamentally, problem spaces and goals exist from the perspective of an observer (a subsystem exploiting a perspective for adaptive advantage). Thus, it is essential to develop an understanding of the dynamics which enable, scale up, and transform the proto-cognitive/computational boundaries of evolved and designed agents.

## Conclusion

"A smooth sea never made a skilled sailor"

— Franklin D. Roosevelt

It is no coincidence that Alan Turing was interested in both the question of intelligence in radically unconventional embodiments [254], and the question of self-organization of biological form during embryogenesis [255]. In a deep sense, they are the same question, seeking to understand the ability of matter to support reprogrammable, software-level (physiological) information processing that provides context-sensitive behavior for changing inputs. Turing’s modeling of self-organization in a chemical medium dovetails with the fact that an egg genome does not specify one embryo in a hardwired manner. Cell sheets that express genetically provided hardware are capable of giving rise to 0, 1, or several autopoietic systems that set their own boundaries in the excitable medium of the early blastoderm [256]—the most basic example of the flexibility of the product of the evolutionary process.

Biological systems exhibit cybernetic competencies in diverse problem spaces, which scale up from the most minimal behaviors of molecular components [257–260] via a process of evolutionary enrichment for computational flexibility and controllability. The fact that the substrate on which evolution works is an agential material [37] means that evolution is searching not only the space of micro-level phenotypic outcomes, but also the smoother, lower-dimensional space of behavior-shaping signals for control of modules that encapsulate complexity behind simple interfaces like the homeostatic control loop. This also allows evolution to bridge the sim-to-real gap—a problem that plagues roboticists and engineers, in which the environment is constantly surprising no matter how rich the prior training set was [261, 262]. In overloading [42] the same genetic hardware with multiple interpretations [125, 126, 197], the physiological machinery of chemical and cellular networks ensures that evolution does not over-train on prior data, but compresses and generalizes. Evolution crosses the gap between prior experience and adaptive novelty [261] by not relying too much on a lineage’s prior experience (which, like our simulations, often fail to recapitulate everything we need to know in the future), but rather by exploiting problem-solving competencies of modules at different scales.

The evolutionary process seems to have been enriched for computational capacity, starting at the level of molecular pathways [88, 89, 263]. It is likely that molecular and tissue-level intelligence is favored for the same reason that nervous systems have been so successful: they enable context-sensitive computation that cannot be pre-specified in the protein-level encoding of the DNA. Biology has many examples of remarkable compression of complex events into molecular representations [264–267], and the converse—cross-scale decoding: these need to be better understood. In fact, even living systems that do not seem to offer much plasticity and whose MCA capacities seem very low (e.g., *C. elegans*) cannot be guaranteed to be as hardwired as they first appear. It is an essential aspect of intelligence that its detection (or lack thereof) says as much about the observer as it does about the system itself [14, 86]; moreover, the vast bulk of research in development and even physiology in model systems has not specifically probed for problem-solving in diverse spaces. It is entirely possible that more sophisticated attempts to uncover such dynamics in novel spaces will yield surprises in model systems that are not known for their MCA capabilities.

How do the molecular events brought on by injecting a frog’s egg with an odorant molecule become expanded into a nervous system architecture which controls muscles to behaviorally seek out that odorant as an adult frog [268]? All of the above suggests that MCA enables evolution to search a simpler, smoother, more powerful space—the space of signals and rewards—rather than the high-dimensional space of individual local hardware states. Examples that support this extend all the way down to the cybernetic properties of chemical pathways, such as learning and habituation [87–89, 269]. By shaping signals that are interpreted by modules with various degrees of memory and decision-making ability, both evolution and engineers can take advantage of the MCA [270]. The ability of biology to move salient information across scales, problem spaces, and mechanistic implementations is likely key to the efficiency and power of evolution.

Homeostatic control cycles may facilitate such scaling of complexity and intelligence because of their functional and semantic modularity: for example, a setpoint can easily be changed from a metabolic (cell-level) metric to a measure of planar polarity alignment (tissue level), while keeping everything else the same. The ability to independently swap out the measurements, comparison function, and setpoints of a homeostatic loop means that a very wide variety of high-level strategies can be readily tested out by evolution. This is the same advantage engineers and end users reap from homeostats: overall function can be rapidly changed without needing many alterations of the underlying hardware by for example rewriting the setpoint (such as when the genetically wild-type planarian’s bioelectric pattern memory is briefly re-set to a two-head pattern by a simple ion channel-targeting stimulus, resulting in a permanent line of worms which reliably build to the new setpoint pattern when fissioning or amputated [194]).

The modulation of the evolutionary process by behavioral intelligence in animals (and science-capable humans) was long predated by a similar process taking place outside the brain: the diverse intelligence of morphogenetic processes operating in anatomical space. The fact that evolution not only finds solutions to specific problems, but also creates somewhat generic problem-solving machines, with multiple diverse (simultaneously existing [42]) capabilities, has many implications for the evolutionary process itself. It facilitates credit assignment for the evolutionary search process, enables exploration and novelty [4, 271], and hugely accelerates the process of increasing complexity. This feedback between evolutionary scaling of intelligence and the acceleration of the discovery of novelty by evolution forms a powerful ratchet [240], which is compatible with the emerging picture of a continuum of basal cognition across the tree of life [14].

## Future directions for research

This perspective suggests a unifying lens for aspects of evolutionary developmental biology and engineering [272], focused on the idea of hacking the agential interfaces of components instead of micro-managing design. Like parasites, body tissues, and evolution itself, bioengineers and roboticists have the opportunity to develop a science of control by exploiting competencies as affordances, applicable equally to the direct products of evolution and to the second-order creative process in which evolved humans partake. Future appliances in this field will be potentiated by universal APIs that go beyond object-oriented programming to goal-oriented programming to exploit biological architectures and offer similar advantages to designed digital systems (as well as hybrid platforms). A number of future research programs can now be pursued.

With respect to theory, much needs to be done to connect these ideas to the body of work on evolutionary units of selection [273, 274], and on natural induction [47]. Tools need to be developed to identify virtual governors [57, 275] as evolutionary forces and to quantify what spaces evolutionary processes are really searching [86]. Computational modeling and synthetic biological systems are now primed to address the dynamics of cognitive scaling and the impacts thereof on the competency of the search process itself, via computational models that explicitly blend low-level competencies with an evolutionary cycle [240, 276]. Some of the biggest questions concern the latent space of homeodynamic goals at different scales, and where these setpoints come from, when not directly specified by genome or environment [271, 272, 277–279]. It is likely that generic principles of inherent laws of biomechanics, bioelectrics, and information processing [62–64, 132, 280–284] will be key parts of the roadmap to flesh out the bi-directional relationship between multiscale intelligence and evolution.

It should be noted that the evolutionary consequences of agential matter were here explored from the perspective of an addition to what is otherwise treated as a fully mainstream picture of the evolutionary process itself. However, in future work, this perspective should be merged with currently developing alternative views of evolution, to determine what implications it has for those frameworks. Other non-traditional [28, 60, 285, 286] evolutionary dynamics could perhaps also be greatly facilitated by the ability of cells to solve novel problems using transcriptional responses**.** One example is the ready fit of these ideas into the emerging framework of natural induction [35, 47], which seeks invariants between the evolutionary process and that of learning broadly conceived. Another has to do with so-called Lamarckian effects [28, 60, 286]. For example, consider the case of regulating the small number of relevant genes to resolve a novel stressor, as in the barium-exposed planaria [90]. This provides clear evidence that cells can sometimes solve the hardest part of reversing the central dogma—the credit assignment needed to know *which genes* to edit to enrich for specific outcomes. Once the relevant transcripts are identified by computational processes at the transcriptional level, making genomic edits at those loci could in principle be done by straightforward, familiar mechanisms (akin to reverse transcriptase, CRISPR, trans-generational chromatin modifications, etc.). Whether this in fact occurs in vivo represents an exciting future direction for experimental research.

## Impacts beyond evolutionary biology

Specific impacts of the above ideas will go far beyond evolutionary biology, for example, to applications in regenerative medicine which will increasingly exploit the decision-making capabilities of cells and tissues, in addition to traditional bottom-up molecular rewiring [160]. Beyond the life sciences, engineering is now heavily reliant on evolutionary approaches to design [287–289], and robust future robotics and AI architectures must mimic the multiscale competency architecture to achieve (and surpass) the functionality of natural systems. Importantly, connectionist AI architectures are not only the beneficiaries of these ideas, but are also a key developing toolkit for the emerging field of basal cognition because of the “impedance match” needed between a phenomenon and the tools used to study it. Physicists use low-agency, mechanical tools to observe the natural world, and inevitably arrive at low-agency mechanistic models. Taking full advantage of virtual governors, proto-cognitive modules, and other aspects of the software of life requires tools that recognize and learn to hack these capacities. Agency cannot be directly observed with a microscope, but brains, evolutionary processes, and emerging machine learning tools are primed to detect and exploit it via agential models of control because they themselves are higher-agency systems.

As foreseen by many workers since the beginnings of thought on evolution, intelligence is key to evolutionary change. Recent consilience of a range of disciplines are giving rise to the field of *diverse intelligence*, which recognizes a spectrum of problem-solving and creative competencies in unconventional, basal media that goes beyond the old dichotomy of “dumb mechanical machine vs. high-level true intelligence”. This finally makes it possible to incorporate a naturalistic, non-reductive, empirically useful conception of intelligence into modern theories of evolutionary change. It also naturally leads to hypotheses that whole lineages, and perhaps even the evolutionary process itself, can be viewed as proto-cognitive, cybernetic agents, by characterizing the competencies of the search dynamics using the language of cognitive and behavioral science such as active inference and Hebbian learning [35, 48, 290–292]. What is becoming increasingly clear is that intelligence is not some latecomer that arrives with the appearance of big brains—it is baked in at the very beginning [293, 294], present at multiple scales of the biological substrate of evolution, and continuously shapes its course in a fluid dance that potentiates all participants.

## Data Availability

N/A.
